# Allopatric and Sympatric Drivers of Speciation in *Alviniconcha* Hydrothermal Vent Snails

**DOI:** 10.1093/molbev/msaa177

**Published:** 2020-07-13

**Authors:** Corinna Breusing, Shannon B Johnson, Verena Tunnicliffe, David A Clague, Robert C Vrijenhoek, Roxanne A Beinart

**Affiliations:** 1 Graduate School of Oceanography, University of Rhode Island, Narragansett, RI; 2 Monterey Bay Aquarium Research Institute, Moss Landing, CA; 3 Department of Biology and School of Earth and Ocean Sciences, University of Victoria, Victoria, BC, Canada

**Keywords:** *Alviniconcha*, speciation, allopatric divergence, ecological isolation, deep-sea hydrothermal vents, chemosynthetic symbionts

## Abstract

Despite significant advances in our understanding of speciation in the marine environment, the mechanisms underlying evolutionary diversification in deep-sea habitats remain poorly investigated. Here, we used multigene molecular clocks and population genetic inferences to examine processes that led to the emergence of the six extant lineages of *Alviniconcha* snails, a key taxon inhabiting deep-sea hydrothermal vents in the Indo-Pacific Ocean. We show that both allopatric divergence through historical vicariance and ecological isolation due to niche segregation contributed to speciation in this genus. The split between the two major *Alviniconcha* clades (separating *A. boucheti* and *A. marisindica* from *A. kojimai*, *A. hessleri*, and *A. strummeri*) probably resulted from tectonic processes leading to geographic separation, whereas the splits between co-occurring species might have been influenced by ecological factors, such as the availability of specific chemosynthetic symbionts. Phylogenetic origin of the sixth species, *Alviniconcha adamantis*, remains uncertain, although its sister position to other extant *Alviniconcha* lineages indicates a possible ancestral relationship. This study lays a foundation for future genomic studies aimed at deciphering the roles of local adaptation, reproductive biology, and host–symbiont compatibility in speciation of these vent-restricted snails.

## Introduction

How novel species evolve in response to environmental pressures remains a fundamental question in evolutionary biology (Schluter 1998). New species can originate through a number of mechanisms, including geographic isolation (allopatric speciation) and formation of reproductive barriers due to localized natural selection (sympatric speciation) (Darwin 1859; Mayr 1942; Dobzhansky 1951; Coyne and Orr 2004; Schuler et al. 2016; Yang et al. 2017). The evolution of new species commonly occurs as a result of ecological or mutation-order speciation (Schluter 2009). Ecological speciation theory predicts that reproductive isolation should evolve between populations adapting to different environments (Mayr 1942) and is a consequence of divergent selection on functional traits enabling specialization for distinct ecological niches (Rundle and Nosil 2005; Schluter 2009). Habitat isolation can occur at different spatial scales that range from “macrospatial” (a form of allopatric speciation, where gene flow between populations is absent due geographic separation) to “microspatial” (where distinct populations co-occur geographically but rarely interbreed because of adaptations to different ecological niches) (Coyne and Orr 2004). By contrast, mutation-order speciation happens when reproductive isolation evolves due to random fixation of alleles in two populations that face the same selective pressures (Mani and Clarke 1990; Schluter 2009; Nosil and Flaxman 2011). Evolutionary divergence under this scenario is unlikely (though not impossible) if gene flow is present (Schluter 2009; Nosil and Flaxman 2011), whereas ecological speciation is common with or without gene flow (Schluter 2009; Nosil and Flaxman 2011; Feder et al. 2012; Campbell et al. 2018). Additional processes including inter-specific hybridization and host–microbe associations have received less attention but are now accepted as significant drivers of speciation (Schuler et al. 2016).

**Fig. 1.**
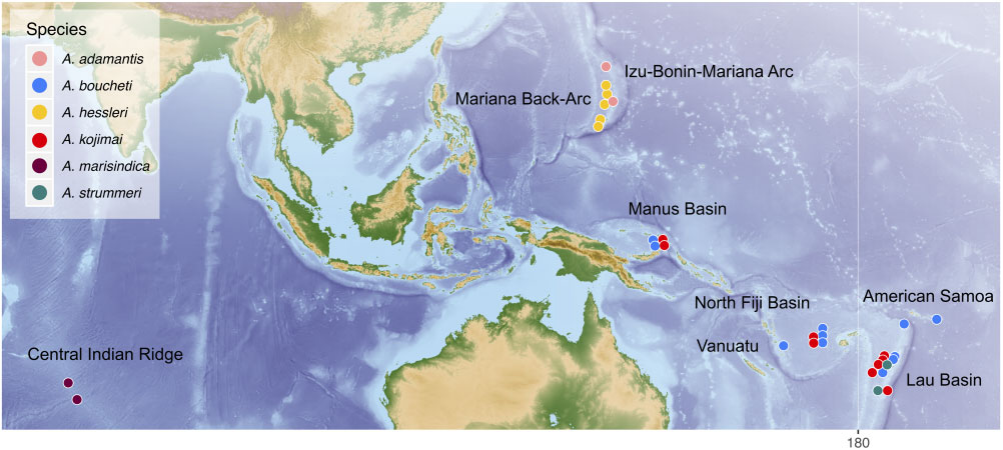
Map of sampling localities for the six *Alviniconcha* species analyzed in this study.

Our current understanding of speciation mechanisms in animals stems mostly from evolutionary research on terrestrial and freshwater organisms, whereas less is known about speciation in oceanic environments, in particular deep ocean basins (Miglietta et al. 2011). Processes that lead to evolutionary divergence in marine systems may differ from other environments given that physical barriers are less pronounced and deep-sea habitats might have been less affected by past climatic fluctuations (Miglietta et al. 2011; but see Vrijenhoek 2013), at least over much of the Cenozoic. Nonetheless, deep-sea hydrothermal vents provide an exception to other deep-sea environments due to their global but island-like distribution and their relative instability on ecological and geological time scales (Vrijenhoek 2013). The dense animal communities living in these habitats are exposed to highly variable and extreme environmental conditions, and many of the invertebrates are sustained through obligate symbiosis with chemosynthetic bacteria (Dubilier et al. 2008). The phylogenetically and ecologically closely related snail genera *Alviniconcha* and *Ifremeria* (Gastropoda: Abyssochrysoidea) are among the dominant inhabitants of Indo-Pacific hydrothermal vents. The genus *Ifremeria* is monotypic and apparently restricted to Western Pacific vents in the Manus, Lau, and North Fiji basins. In contrast, the broadly distributed genus *Alviniconcha* comprises six species (Johnson et al. 2015): *Alviniconcha kojimai* and *Alviniconcha boucheti* co-occur in the Manus, Lau, and North Fiji basins; *Alviniconcha strummeri* is known from the southern Lau Basin; *Alviniconcha hessleri* and *Alviniconcha adamantis* appear to be endemic to the Mariana Back-Arc and Volcanic Arc, respectively; and *Alviniconcha marisindica* occurs along the Central Indian Ridge (fig. 1 and table 1).

**Table 1.** Geographic Coordinates, Depths, Years of Sampling, and Number of Samples for Each Vent Site Investigated in This Study.

Dive #	Locality	Abbr.	Latitude	Longitude	Depth (m)	Year
	American Samoa					
H-1773	Vailulu’u Seamount		–14°12.610′	–169°02.776′	678	2019
	Lau Basin					
R-1918/1919	Niua South	NS	–15°09.885′	–173°34.468′	1,156–1,164	2016
J2-140/141	Kilo Moana	KM	–20°03.222′	–176°08.009′	2,620	2005
J2-142	Tow Cam	TC	–20°19.076′	–176°08.258′	2,714	2005
R-1933			–20°19.000′	–176°08.204′	2,705	2016
R-1932/1935	Tahi Moana	THM	–20°40.409′	–176°10.848′	2,273–2,280	2016
R-1922/1931	ABE	ABE	–20°45.700′	–176°11.500′	2,130–2,155	2016
J2-143/144	Tu’i Malila	TM	–21°59.431′	–176°34.146′	1,845	2005
R-1924–1930			–21°59.347′	–176°34.092′	1,883–1,889	2016
	North Fiji Basin					
J2-149/150	White Lady	WL	–16°59.398′	173°54.953′	1,970	2005
J2-151/152	Mussel Hill	MH	–16°59.410′	173°54.970′	1,973	2005
J2-153	White Rhino	WR	–16°59.446′	173°54.862′	1,978	2005
	Mariana Back-Arc					
J2-42	Snail Site	SN	12°57.250′	143°37.200′	2,863	2003
J2-185/S-185	Forecast	FC	13°23.680′	143°55.207′	1,447–1,475	2006
Su-47	Perseverance		15°28.810′	144°30.462′	3,909	2016
Su-41/44	Hafa Adai		16°57.669′	144°52.017′	3,274–3,278	2016
Su-40	Burke		18°10.954′	144°43.193′	3,631	2016
Su-39	Alice Main		18°12.619′	144°42.438′	3,611	2016
S-140/141	Alice Springs		18°12.800′	144°42.400′	3,589	1992
Su-37	Illium		18°12.815′	144°42.450′	3,582	2016
	Izu-Bonin-Mariana Arc					
R-787	E. Diamante Seamount	EDS	15°56.570′	145°40.880′	351–357	2004
J2-193						
Su-36	Chamorro Seamount		20°49.287′	144°42.423′	920	2016
	Manus Basin					
MQ-309	Roman Ruins		–3°43.238′	151°40.519′	1,685	2011
MQ-306	Snow Cap		–3°43.684′	151°40.158′	1,647	2011
ST-28/30	Solwara 8-2	SW8	–3°43.824′	151°40.458′	1,710	2008
ST-9	Solwara 1-4	SW1	–3°47.436′	152°05.472′	1,530	2008
ST-11	Solwara 1-5	SW1	–3°47.370′	152°05.778′	1,490	2008
ST-17	Solwara 1-6	SW1	–3°47.370′	152°05.616′	1,480	2008
MQ-314	North Su		–3°47.965′	152°06.089′	1,187–1,199	2011
MQ-316						
ST-38	South Su-7	SSU	–3°48.564′	152°06.144′	1,300	2008
ST-40	South Su-8	SSU	–3°48.492′	152°06.186′	1,350	2008
	Vanuatu	VA				
KI-27/60	Nifonea		–18°08.000′	169°31.000′	1,900	2013
	Central Indian Ridge					
J2-301	Edmunds	ED	–23°31.800′	69°21.600′	3,289	2001
J2-297	Kairei	KA	–25°52.980′	70°35.820′	2,432	2001

Note.—Submersibles: H, Hercules; J2, Jason II; S, Shinkai 6500; Su, SuBastian; R, Ropos; ST, ST212; KI, Kiel 6000; MQ, Marum Quest.

*Alviniconcha* and *Ifremeria* are nutritionally dependent on horizontally transmitted, chemosynthetic sulfide-oxidizing endosymbionts that are acquired from a diverse community of environmental bacteria and are harbored within vacuoles in the gill filaments (Windoffer and Giere 1997; Suzuki, Sasaki, Suzuki, Nogi, et al. 2006; Trembath-Reichert et al. 2019). Except for *A. boucheti* and *A. marisindica*, which are constrained to symbionts of the class Campylobacteria (formerly Epsilonproteobacteria; Waite et al. 2017), all other *Alviniconcha* species and *Ifremeria nautilei* typically host Gammaproteobacteria symbionts (Urakawa et al. 2005; Suzuki, Sasaki, Suzuki, Nogi, et al. 2005; Suzuki, Sasaki, Suzuki, Tsuchida, et al. 2005; Suzuki, Kojima, Sasaki, et al. 2006; Suzuki, Kojima, Watanabe, et al. 2006; Beinart et al. 2012). In zones of sympatry, the two snail genera occupy relatively narrow ranges of chemical and thermal conditions that are well within their physiological tolerance limits and that do not support maximal rates for chemoautotrophic growth (Sen et al. 2013). This observation suggests that interspecies competition is an important factor determining the realized niche of these snails (Sen et al. 2013)*.*Beinart et al. (2012) proposed that host niche utilization is strongly influenced by the availability and metabolic capacity of locally dominant symbiont phylotypes. In their study of co-occurring *Alviniconcha* species from the Lau Basin, they showed that the distribution and abundance of host–symbiont combinations (holobionts) followed a latitudinal gradient in vent geochemistry, wherein *A. boucheti* holobionts dominated at northern localities characterized by high end-member concentrations of sulfide and hydrogen (∼3.5–7 mM and ∼100–500 µM, respectively), *A. kojimai* holobionts dominated at midlatitude localities characterized by medium end-member concentrations (∼2.5–4 mM and ∼50–100 µM, respectively), and *A. strummeri* holobionts dominated at southern localities characterized by low end-member concentrations of these reductants (∼1–3 mM and 35–135 µM, respectively). These broad-scale patterns of microbe-associated habitat segregation suggest that the endosymbionts might have played a significant role in the diversification of these gastropods.

Despite their phylogenetic and ecological affinities, *Ifremeria* and *Alviniconcha* have undergone remarkably different degrees of speciation. In the present study, we used fossil-calibrated molecular phylogenies and population genetic analyses based on mitochondrial and nuclear markers to obtain insights into allopatric and sympatric mechanisms that led to the high degree of diversification in the genus *Alviniconcha*. To assess the role of allopatric isolation, we compared the phylogenetic divergence events among the Abyssochrysoidea with Mesozoic and Cenozoic geological events of the Indo-Pacific Ocean basins and examined genetic subdivision within several broadly distributed species. To determine evidence for sympatric isolation, we compared the time-calibrated phylogenies with the diversity and phylogenetic relationships of the snails’ chemosynthetic endosymbionts and assessed the potential for hybridization among co-occurring *Alviniconcha* species.

## Results

### Gene Networks

**Fig. 2.**
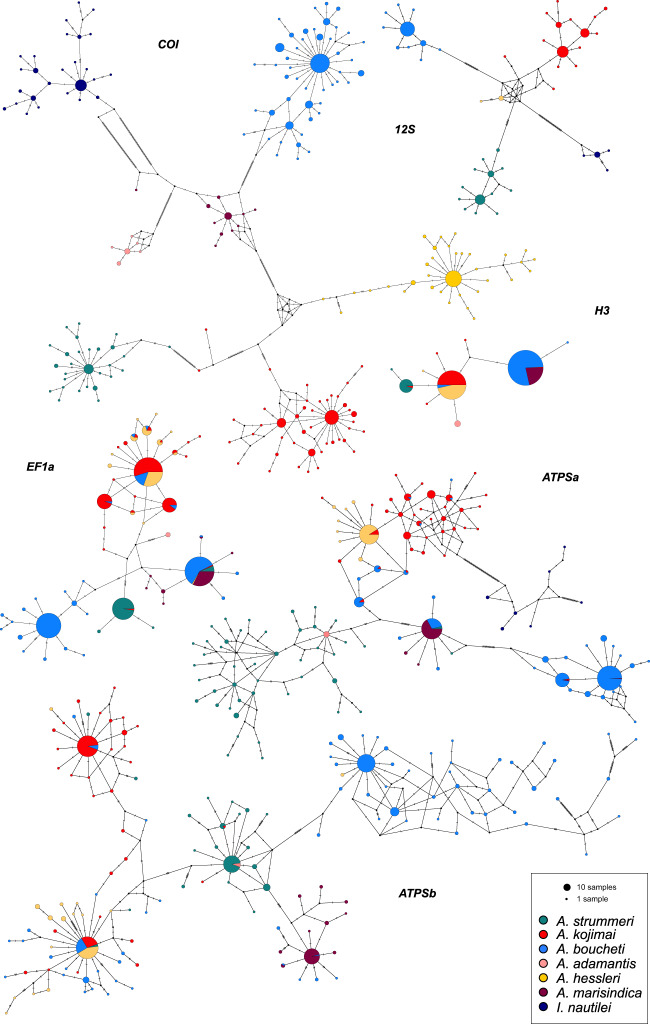
Haplotype networks for the two mitochondrial (*COI* and *12S*) and four nuclear genes (*H3*, *EF1a*, *ATPSa*, and *ATPSb*) that were used for population genetic analyses. Circles represent individual haplotypes, where circle size is proportional to haplotype frequency and black circles indicate unsampled haplotypes. Dashes on branches denote the number of mutations between haplotypes.

Haplotype networks for the two mitochondrial genes, *COI* and *12S*, indicated complete sorting among lineages for the six *Alviniconcha* species and their sister taxon *I. nautilei* (fig. 2), as previously reported (Johnson et al. 2015). The *COI* sequences from *Alviniconcha* and *Ifremeria* differed by at least 75 mutations, whereas pairs of *Alviniconcha* species differed by 25–48 mutations. The smallest divergence was observed between *A. kojimai* and *A. hessleri* and the largest between *A. strummeri* and *A. adamantis*. Sequence divergence was lower for the *12S* locus, with a maximum of 19 substitutions between *Alviniconcha* species pairs (fig. 2). The nuclear markers generally confirmed species identities as seen with the mitochondrial loci, although they were less diagnostic due to varying degrees of incomplete lineage sorting and smaller divergence between sequences (fig. 2). Shared polymorphisms were particularly evident between *A. kojimai* and *A. hessleri,* as well as between *A. boucheti* and *A. marisindica*, whereas *A. strummeri* and *A. adamantis* had higher amounts of private alleles.

### Population Structure and Admixture

**Fig. 3.**
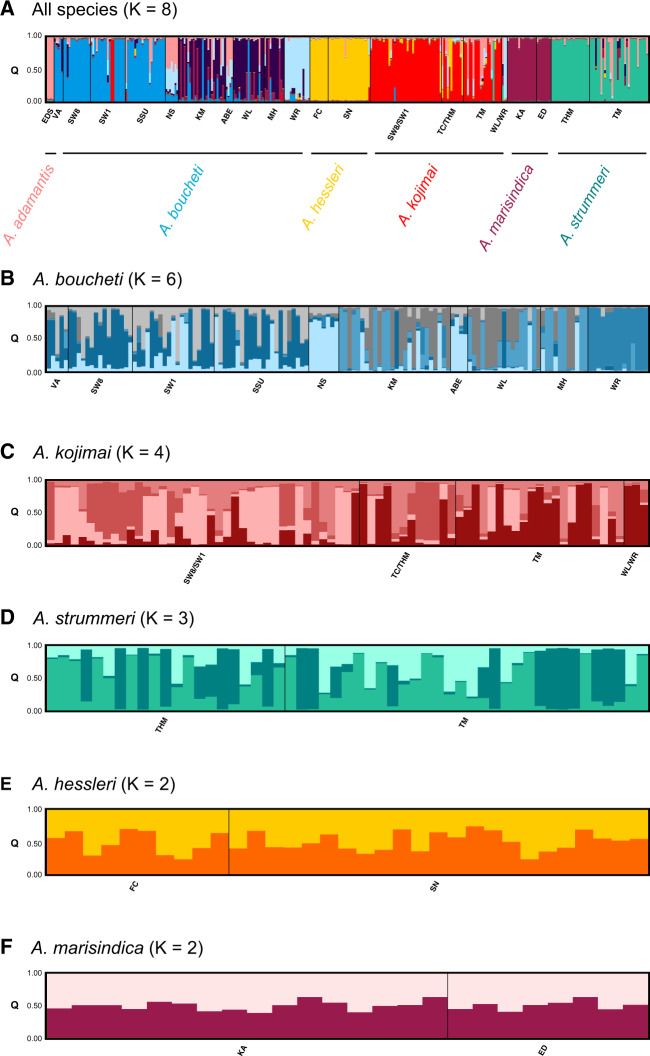
Genetic structure of *Alviniconcha* species (*A*) and populations (*B–F*). Each color represents a different genetic cluster (*K*), whereas each vertical bar represents a distinct individual, where colored line segments are proportional to genetic group memberships. Black lines divide individuals from distinct sampling locations noted along the *x*-axis. Lau Basin sites: KM, Kilo Moana; TC, Tow Cam; TM, Tu’i Malila. North Fiji Basin sites: WL, White Lady; MH, Mussel Hill; WR, White Rhino. Manus Basin sites: SW8,  Solwara 8; SW1,  Solwara 1; SSU, South Su; VA, Vanuatu. Mariana Back-Arc sites: FC, Forecast; SN, Snail Site; EDS, E. Diamante Seamount. Central Indian Ridge sites: KA, Kairei; ED, Edmunds.

Structure analyses distinguished the six previously described *Alviniconcha* species (Johnson et al. 2015) based on mitochondrial and nuclear markers (fig. 3 and supplementary fig. S1, Supplementary Material online). Runs including the mitochondrial *COI* gene provided in general higher resolution of population genetic subdivision than runs based on nuclear loci alone (supplementary figs. S1–S6, Supplementary Material online). Therefore, we report results from the combined marker set in the manuscript, but Structure plots for all *K* values and marker combinations can be found in the supplementary figures S1–S6, Supplementary Material online. Although the most parsimonious grouping following the method by Evanno et al. (2005) was *K *=* *2, visual inspections of higher *K* values (Meirmans 2015) showed clear distinctions of up to eight clusters, indicating population structure within the sympatric species *A. boucheti*, *A. kojimai*, and *A. strummeri* (fig. 3 and supplementary figs. S1–S4, Supplementary Material online)*.* For all three species, significant genetic variation among individuals within vent localities was observed (fig. 3).

*Alviniconcha boucheti* comprised six genetically connected clusters that were broadly partitioned across four geographic regions: 1) Vanuatu and Manus Basin, 2) Kilo Moana (Lau Basin), White Lady, and Mussel Hill (North Fiji Basin), 3) Niua South and ABE (Lau Basin), and 4) White Rhino (North Fiji Basin). *Alviniconcha kojimai* was represented by four genetic clusters that were subdivided across the 1) Manus Basin and 2) North Fiji Basin, with Lau Basin populations showing similarities to both of these groups. *Alviniconcha strummeri* exhibited less genetic partitioning, with the Tu’i Malila locality slightly differentiated from the Tahi Moana locality in the Lau Basin. Populations of *A. hessleri* and *A. marisindica* were not structured across the sampled geographic regions, but limited genetic differences were present among individuals (fig. 3 and supplementary figs. S5 and S6, Supplementary Material online). Despite the presence of shared polymorphisms, ABBA–BABA tests provided no evidence for hybridization between sympatric species, indicating that patterns of shared genetic variation were due to incomplete lineage sorting (table 2). Note, however, that *D* values for the first ABBA–BABA test were close to –1, and it is possible that gene flow between *A. kojimai* and *A. boucheti* might have been detected with a larger genomic marker set.

**Table 2.** Results from ABBA–BABA Tests (*D* statistics).

Outgroup	P3	P2	P1	# ABBA	# BABA	*D*	*Z*	*P* Value
*A. adamantis*	*A. boucheti*	*A. strummeri*	*A. kojimai*	11	20	–0.93	NA	0.33
*A. boucheti*	*A. strummeri*	*A. hessleri*	*A. kojimai*	0	4	NA	NA	0.95

Note.—ABBA and BABA sites are parsimony informative sites that result in discordances between gene trees and species trees due to the presence of incomplete lineage sorting or gene flow. ABBA and BABA sites are expected to be equally frequent under incomplete lineage sorting but will have uneven abundances if gene flow is present. *D* = Patterson’s *D* estimate based on Jackknife resampling. A significant *D *>* *0 indicates gene flow between P3 and P2, whereas a significant *D *<* *0 indicates gene flow between P3 and P1. *P* values computed by Fisher’s combined probability test were not significant. *Z *=* Z* score of Patterson’s *D*.

### Fossil-Calibrated Phylogeny and Species Tree

**Fig. 4.**
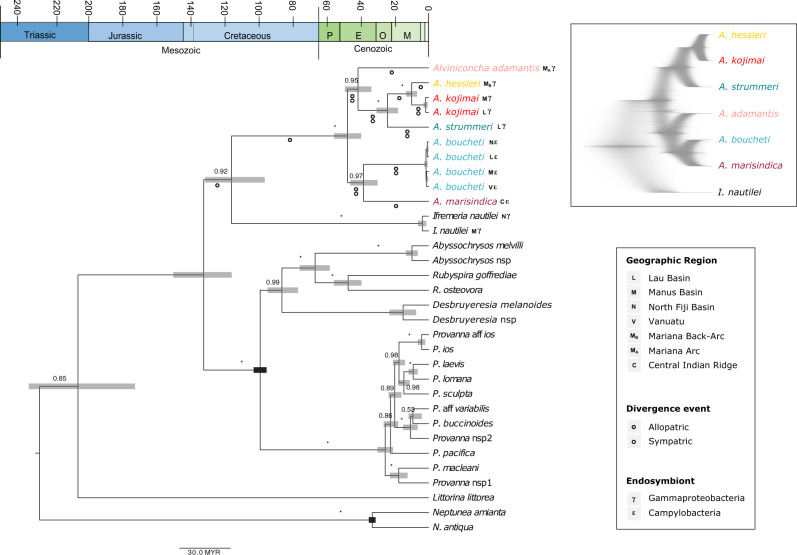
Fossil-calibrated phylogeny of the Abyssochrysidae. Scale bar is 30 Myr, stars on branches indicate 1.0 support value. Gray bars indicate the highest posterior probability density range for estimates of lineage splitting times. The legend provides symbol codes for the geographic distribution, endosymbiont affinity, and putative sympatric (white circle) and allopatric (gray circle) divergence events of the different *Alviniconcha* lineages. If both white and gray circles are shown under a branch, both allopatric and sympatric processes might have contributed to the respective divergence event. Inset in the upper right shows the species tree obtained with StarBeast, with gray shading indicating uncertainties in the phylogenetic placements.

Fossil-calibrated Beast analyses based on ribosomal, mitochondrial, and histone marker genes revealed that the Abyssochrysidae is a relatively old family (fig. 4), having diverged from the Littorinidae and Buccinidae in the early Jurassic ∼200 Ma (95% Highest Posterior Density (HPD): 172–234 Ma). This range agrees well with the divergence times reported by Johnson et al. (2010) (93–228 Ma) but is slightly older than estimates by Osca et al. (2014) (144–168 Ma). The split between *I. nautilei* and *Alviniconcha* spp. occurred in the mid-Cretaceous ∼113 Ma (95% HPD: 96–131 Ma). The extant *Alviniconcha* lineages began to diversify in the Eocene ∼48 Ma (95% HPD: 39–56 Ma) and comprised two well-supported clades plus *A. adamantis*, which formed a basal, unresolved branch that split ∼42 Ma (95% HPD: 34–49 Ma). The first well-supported clade included *A. strummeri*, *A. kojimai*, and *A. hessleri* and diverged ∼25 Ma (95% HPD: 18–31 Ma). The second clade included *A. marisindica* and *A. boucheti* with a divergence time of ∼38 Ma (95% HPD: 30–46 Ma). The most recent split occurred between *A. kojimai* and *A. hessleri* in the later Miocene ∼10 Ma (95% HPD: 7–13 Ma).

In general, the topology of the species tree for *Alviniconcha* agreed with that of the concatenated gene tree, although the position of *A. adamantis* indicated a potential sister relationship to *A. boucheti* and *A. marisindica* (fig. 4). The second species tree including the nuclear markers *EF1a*, *ATPSb*, and *ATPSa* resulted in an unresolved polytomy and we were unable to obtain robust estimates for phylogenetic placements (supplementary fig. S7, Supplementary Material online).

### Phylogenetics of Endosymbionts

**Fig. 5.**
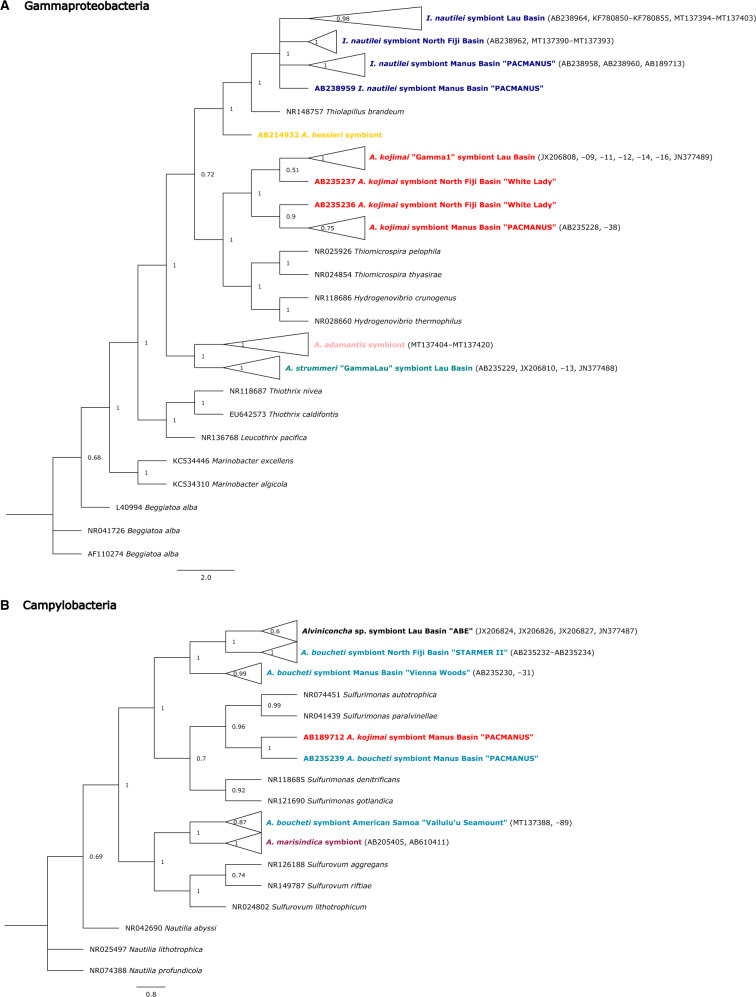
Phylogenies of the (*A*) Gammaproteobacteria and (*B*) Campylobacteria symbiont lineages. Node labels indicate node support probabilities, whereas scale bars show the expected number of substitutions per nucleotide site. Color codes are based on host species identities as shown in figures 1 and 2.

Molecular Bayesian analyses of the full-length *16S* rRNA revealed geographical partitioning for the endosymbionts of *Alviniconcha* and *Ifremeria*, suggesting that symbiont phylotypes associated with *I. nautilei*, *A. boucheti*, and *A. kojimai* were genetically distinct between the Lau, Manus, and North Fiji basins (fig. 5)*.* The endosymbionts of *I. nautilei* were most closely related to free-living *Thiolapillus brandeum* and the Gammaproteobacteria symbiont of *A. hessleri.* Together, these bacterial groups formed a neighboring clade to the “γ-1” endosymbionts of *A. kojimai* (Beinart et al. 2012) and free-living *Thiomicrospira* and *Hydrogenovibrio* (fig. 5*A*). In both the Lau and Manus basins, *A. kojimai* additionally hosted Campylobacteria lineages that were also found in *A. boucheti*. The endosymbionts of *A. adamantis* formed a distinct clade that was most closely related to the “γ-Lau” group present in *A. strummeri* (Beinart et al. 2012).

*Alviniconcha marisindica* and *A. boucheti* were the only species that harbored predominantly Campylobacteria symbionts (fig. 5*B*). Although *A. marisindica* contained a single *16S* rRNA phylotype related to free-living *Sulfurovum*, *A. boucheti* associated with distinct Campylobacteria lineages that were related to free-living *Sulfurimonas*, with one exception: *A. boucheti* from American Samoa harbored a phylotype that was most closely related to the *A. marisindica* symbiont.

**Fig. 6.**
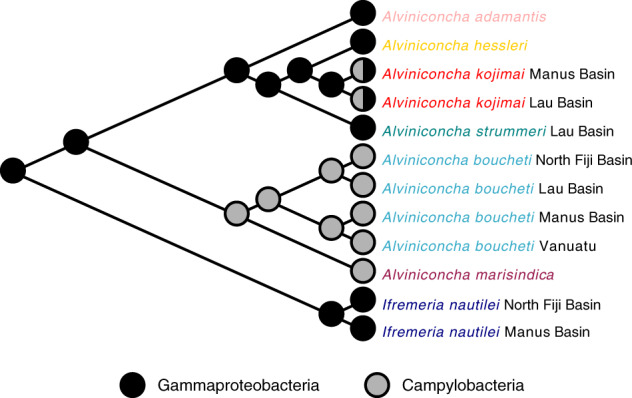
Analysis of endosymbiont trait evolution in *Alviniconcha*. Associations with Gammaproteobacteria lineages represent the ancestral condition, whereas the Campylobacteria endosymbiosis is a derived state.

Character traces of symbiont type identified the Gammaproteobacteria endosymbiosis as the ancestral condition given that the evolutionary older host species harbored only Gammaproteobacteria (fig. 6). By contrast, the Campylobacteria endosymbiosis appears to be a derived trait that evolved multiple times independently.

### Symbiont Phylotypic Diversity and Partitioning

Amplicon sequencing of the *16S V4–V5* hypervariable regions generated an average of 21,302 paired-end reads/sample. These were reduced to ∼9,613 reads/sample after read merging. Sequence denoising resulted in 85 zero-radius operational taxonomic units (ZOTUs), 11 of which were verified as *Alviniconcha* or *Ifremeria* endosymbionts. Oligotyping of amplicon reads comprising these symbiont ZOTUs identified a total of 23 symbiont *16S* rRNA oligotypes in the data set. For 337 snail samples, we were able to obtain both host species identities and *16S* oligotypes. Only these individuals were, therefore, used for further analysis (supplementary table S1, Supplementary Material online).

**Fig. 7.**
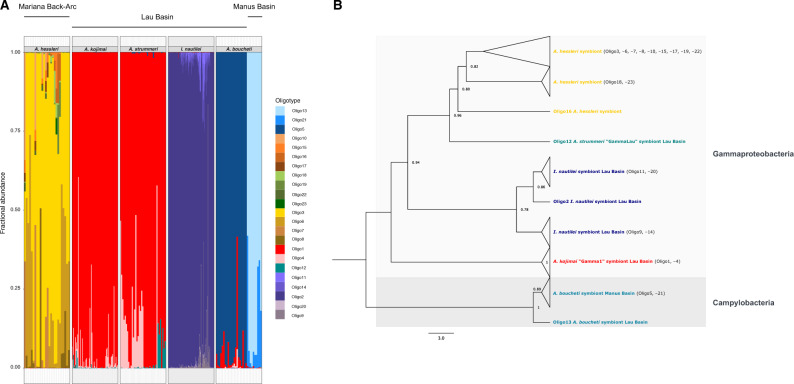
(*A*) Fractional abundances and (*B*) phylogenetic relationships of symbiont *16S V4–V5* oligotypes in *Alviniconcha* (163 specimens) and *Ifremeria* (173 specimens).

**Fig. 8.**
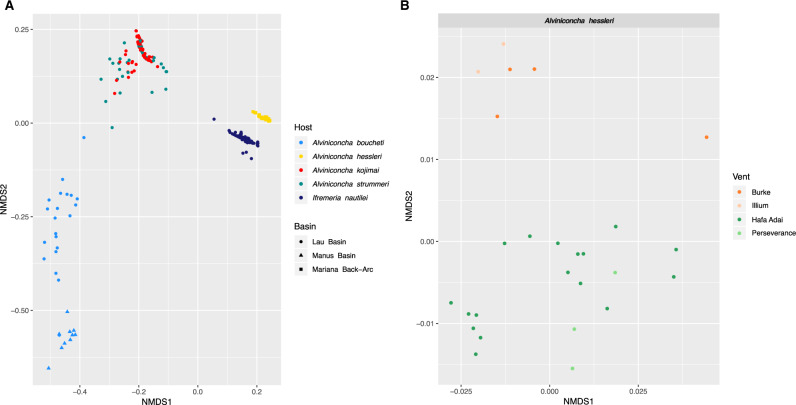
(*A*) Nonmetric dimensional scaling plot of the symbiont *16S* phylotypes based on weighted Unifrac distances. The plot shows that the symbiont composition correlates with the type of host species and is partitioned across back-arc basins for *Alviniconcha boucheti*. (*B*) Detailed nonmetric dimensional scaling plots for symbiont phylotypes associated with *Alviniconcha hessleri*. Symbiont populations form two latitudinal clusters corresponding to ∼16°N (Hafa Adai, Perseverance) and ∼18°N (Burke, Illium).

Each host individual contained one majority symbiont phylotype together with up to 12 minority variants, with *A. hessleri* exhibiting the highest and *A. boucheti*, *A. kojimai*, and *A. strummeri* exhibiting the lowest symbiont richness (fig. 7). In general, symbiont phylotypes corresponded to host species, except in the case of *A. kojimai* and *A. strummeri*, where symbiont compositions largely overlapped (figs. 7 and 8). All host species, with the exception of *A. boucheti,* comprised mostly a combination of different Gammaproteobacteria lineages. *Alviniconcha boucheti* was dominated by Campylobacteria symbiont phylotypes, although in the Lau Basin this species also harbored Gammaproteobacteria symbionts that were typically associated with *A. kojimai* and *A. strummeri*. However, due to presence of the *A. kojimai* symbiont (Oligo1) in negative controls on the same well plate where these *A. boucheti* samples were processed, we cannot exclude the possibility that some of these observations resulted from well-to-well contamination. In accordance with our phylogenetic analyses, the symbionts associated with *A. boucheti* were partitioned across basins. In most cases, we did not have sufficient sample sizes to reveal potential within-basin structuring of the Gammaproteobacteria or Campylobacteria symbiont phylotypes, with the exception of the *A. hessleri* symbionts, which seemed to form two latitudinal clusters (fig. 8).

Partial Mantel tests supported the described patterns, indicating significant correlations between symbiont and host genetic distances (*r *=* *0.76; *P *=* *1e-04) as well as between symbiont genetic distance and geographic distance (*r *=* *0.55; *P *=* *1e-04). By contrast, no association between host genetic distance and geographic distance was found.

## Discussion

In the present study, we performed fossil-calibrated phylogenetic reconstructions and population genetic analyses to illuminate mechanisms of speciation in provannid snails of the genus *Alviniconcha*, which are dominant members of deep-sea hydrothermal vent ecosystems in the Western Pacific and Indian Ocean.

Major tectonic and climatic changes in the Indo-Pacific during the Mesozoic and Cenozoic probably played a central role for geographic isolation during the early evolution of *Alviniconcha* snails. Our fossil-calibrated phylogeny indicates that *Ifremeria* and *Alviniconcha* split 96–131 Ma close to the peak of the Aptian oceanic anoxic event (Li et al. 2008). Although anoxic events led to widespread declines of marine biodiversity, they also created novel opportunities for habitat specialization and allopatric speciation within oxygen-depleted deep-sea environments, such as hydrothermal vents (Rogers 2000). Similarly, expansion of oxygen minimum zones might have contributed to the evolution of the most recent common ancestor of extant *Alviniconcha*, which originated during the upper Eocene (∼50 Ma) shortly after the Paleocene–Eocene Thermal Maximum, another period that was characterized by increasing anoxia in the deep sea. Around the same time, the Philippine Sea Plate developed above the Manus hotspot, and a wide deep-water passage connected the Indian and Pacific Oceans (Hall et al. 1995; Hall 2002; see figs. 14–25 in Hall 2002). This passage started to narrow around 30 Ma and eventually closed 25 Ma, when the Australian Plate subducted under the Philippine Sea Plate and created physical barriers to dispersal (Gaina and Müller 2007).

Gradual vanishing of the Indo-Pacific deep-water connection is consistent with the timing of the most recent common ancestors of the two major *Alviniconcha* clades and likely promoted their diversification: 1) *A. marisindica* and *A. boucheti* (∼30–46 Ma) and 2) *A. hessleri*, *A. kojimai*, and *A. strummeri* (∼18–31 Ma). A variety of along-arc splitting and rifting events in the Miocene to Pliocene subsequently led to the formation of the North Fiji Basin (∼12 Ma), Mariana Back-Arc Basin (∼7 Ma), and Lau Basin (∼5 Ma) (Hall 2002; Crawford et al. 2003; Straub et al. 2015). Evolution of the North Fiji Basin and Mariana Back-Arc Basin corresponds well with the divergence of *A. hessleri* and *A. kojimai* (∼10 Ma), suggesting a potentially allopatric origin of these lineages that might have allowed *A. hessleri* to invade deep-water vent sites of the Mariana Back-Arc, where it is nowadays an endemic species. *Alviniconcha adamantis* is the only other *Alviniconcha* species that occurs in the Mariana region, but it is restricted to shallow vents on volcanoes of the Izu-Bonin-Mariana Arc, which have been active in some form for 40 My (Stern et al. 2003). Interestingly, the phylogenetic position of *A. adamantis* was not well supported and formed a basal branch to the two other clades of *Alviniconcha*. Although we do not have enough data to determine its placement, the *A. adamantis* lineage might be ancestral to other *Alviniconcha* lineages. If true, this genus might have followed a gradual transition from shallow to deep-water vent sites, as has been proposed for bathymodiolin mussels (e.g., Jones et al. 2006; Miyazaki et al. 2010; but see Thubaut et al. 2013).

Although our results suggest that splits between some *Alviniconcha* species involved geographic subdivision, allopatric divergence alone might be insufficient to explain all instances of speciation. For example, *A. boucheti*, *A. kojimai*, and *A. strummeri* live sympatrically in the Lau Basin, and *A. boucheti* and *A. kojimai* also coinhabit vents in the North Fiji and Manus basins. For both of these species, genetic structure analyses indicated varying degrees of genetic subdivision not only among but also within disparate back-arc basins. However, based on recent biophysical models for the Western Pacific region (Mitarai et al. 2016), ocean circulation should support within-basin connectivity, especially for species that are assumed to have planktotrophic larvae with high dispersal potentials, such as *Alviniconcha* (Warén and Bouchet 1993). These contrasting findings suggest that antagonistic ecological factors might exert strong selection against immigrants with maladapted phenotypes, thereby counterbalancing the homogenizing effect of dispersal and allowing speciation in the presence of gene flow. The present genetic data do not allow us to confidently distinguish whether the observed speciation patterns occurred during primary sympatry or resulted from secondary colonization following allopatry. At least the ancestors of *A. kojimai* and *A. strummeri* likely co-occurred in the predecessor of the modern Manus Basin and, thus, may have evolved sympatrically. Nevertheless, opening of the younger Lau and North Fiji basins could have allowed for brief geographic separation (<5–10 Ma), if species invaded these regions asynchronously.

Hydrothermal vents are environmentally dynamic habitats that offer ample opportunities for niche segregation along steep thermal and geochemical gradients. Geochemical patchiness and the availability and/or fitness of symbiont phylotypes (rather than functional host anatomy) are believed to affect spatial partitioning among *Alviniconcha* species in the Eastern Lau Spreading Center (Beinart et al. 2012; Laming et al. 2020). *Alviniconcha boucheti*, which typically hosts Campylobacteria symbionts, was most abundant at northern vent localities characterized by high sulfide and hydrogen concentrations. On the other hand, *A. kojimai* and *A. strummeri*, which usually host Gammaproteobacteria symbionts, were more frequent at southern vent localities with lower sulfide and hydrogen concentrations. Functional differences among the symbiont types combined with genetically based host–symbiont specificities could explain the broad-scale segregated distribution of these species and might have provided a mechanism for their diversification. Free-living chemosynthetic Campylobacteria are known to encode sulfide-oxidizing enzymes that are most effective at high H_2_S concentrations (Han and Perner 2015), providing a selective advantage over Gammaproteobacteria at sulfide-rich vents (Nakagawa and Takai 2008; Yamamoto and Takai 2011; Patwardhan et al. 2018). Recent physiological experiments and transcriptomic analyses imply that this might also be the case for the Lau Basin *Alviniconcha* symbionts (Breusing et al. 2020). Possible relationships between vent geochemistry, symbiont phylotypes, and host species have not been similarly explored in other back-arc basins. Nevertheless, the PACMANUS and Vienna Woods vent fields in the Manus Basin are known to exhibit physicochemical gradients similar to those found in the Lau Basin (Reeves et al. 2011), providing comparable opportunities for symbiont-related niche differentiation. Our partial Mantel tests and NMDS analyses involving *Alviniconcha* species from the Lau and Manus basins, and the Mariana Back-Arc revealed a significant association of symbiont genotypes with host species and vent localities, suggesting that symbiont-mediated habitat segregation might be common in chemosynthetic vent snail symbioses.

Together, our host phylogenetic and bacterial phylotype analyses imply a possible role for endosymbionts in *Alviniconcha* speciation. For instance, *A. boucheti* and *A. marisindica* comprise a monophyletic clade associated predominantly with Campylobacteria symbionts. A trace of character trait evolution indicated that Campylobacteria endosymbionts were acquired in the most recent common ancestor of these two host species, possibly prior to geological events that contributed to splitting of the *A. boucheti*–*A. marisindica* clade. It is possible that a switch to a new symbiont type offered novel ecological opportunities, thereby promoting adaptive divergence from the Gammaproteobacteria-dominated *Alviniconcha* lineages. Both *A. boucheti* and *A. marisindica* associate with symbionts related to free-living *Sulfurovum*, whereas *A. boucheti* also associates with symbionts of the genus *Sulfurimonas*, indicating that the *Sulfurovum* endosymbiosis might be the evolutionarily older condition in these two host species. Speciation due to symbiont-driven niche expansion has been proposed for deep-sea mytilid mussels, with similar time intervals of diversification as observed for provannid snails (Lorion et al. 2013). Interestingly, geographic partitioning of the *A. boucheti* symbiont phylotypes corresponded broadly with the genetic structure of the host populations. Although we cannot exclude the hypothesis that these covarying patterns were mediated by geographic separation, gene flow between basins is expected on evolutionary time scales (Mitarai et al. 2016); therefore, genetically based host–symbiont specificities might also shape the diversification of both partners.

Presently, no substantive evidence exists for past hybridization between broadly co-occurring *A. boucheti* and *A. kojimai* (although future genomic studies may provide other insights into this question). Host–symbiont incompatibilities might contribute to hybrid dysgenesis, a scenario that has been described in insect–microbe symbioses (e.g., Feder et al. 1994; Brucker and Bordenstein 2012, 2013). Although *A. kojimai* occurs in association with Campylobacteria symbionts, these observations were relatively rare, indicating a bias toward a Gammaproteobacteria endosymbiosis (Beinart et al. 2012). Microbial symbionts are currently considered the most disregarded element in animal and plant evolution (Shropshire and Bordenstein 2016). A recent study has shown that both transmission mode and symbiont function influence the degree of host dependence that can ultimately lead to major evolutionary transitions and innovations (Fisher et al. 2017). Although strong host dependence is usually correlated with vertical transmission mode, the same effect can be achieved in horizontally transmitted symbioses if symbionts provide an essential nutritional benefit (Fisher et al. 2017). Consequently, although further studies are needed to characterize the role of symbionts in host speciation, their impact is likely of particular significance in chemo- and photosynthetic ecosystems where host fitness is tightly linked to symbiont metabolism, such as hydrothermal vents, cold seeps, and coral reefs.

An alternative, though not mutually exclusive, scenario for sympatric speciation in *Alviniconcha* could be rapid reproductive isolation and sexual selection. In many organisms, including marine snails of the genera *Haliotis* and *Tegula*, egg and sperm proteins are under strong positive selection and evolve at an accelerated rate that could ultimately split populations into reproductively isolated species (Galindo et al. 2003; Panhuis et al. 2006; Hellberg et al. 2012). Subsequent inter-specific gene flow could be reduced further due to assortative mating, which is often observed in gastropods with internal fertilization (Johannesson et al. 2008; Zahradnik et al. 2008). Although little is known about the reproductive biology of Provannidae, internal fertilization is the implied reproductive mode in this family (Gustafson and Lutz 1994).

Our study suggests that both geographical and ecological divergence (followed by putative reproductive isolation) played significant roles in the evolutionary diversification of the six extant *Alviniconcha* lineages at Indo-Pacific hydrothermal vents. It remains to be determined why the same phenomena did not similarly affect speciation within the genus *Ifremeria*, which is represented by only a single species. The bounded hypothesis on diversification proposes that the evolution of new species is constrained by competition for available niche space (e.g., Rabosky 2009; Price et al. 2014; Larcombe et al. 2018) and that an increase in the number of species within one clade may be compensated by a decline in another (Ricklefs 2010). Given that *Ifremeria* has similar ecological requirements as *Alviniconcha* (Podowski et al. 2010), it is possible that competitive exclusion and niche filling might have limited diversification within this genus. Future genomic and ecological studies may be helpful to illuminate some of these questions and to assess the relative importance of host–symbiont incompatibilities, local adaptation, and reproductive barriers in the evolutionary history of these gastropods.

## Materials and Methods

### Sample Collection and DNA Methods

Snail samples were collected from 32 localities in the Western Pacific and Indian Oceans (table 1). DNA extraction, general polymerase chain reaction (PCR) conditions, amplicon purification, and DNA sequencing used methods that were reported for provannid snails (Johnson et al. 2010, 2015). Twelve primer sets were used to amplify three mitochondrial (*COI*, *mt-12S*, and *mt-16S*) and seven nuclear (*H3*, *28S-D1*, *28S-D6*, *18S*, *ATPSa*, *ATPSb*, and *EF1a*) DNA targets from vent snail hosts as well as the *16S* rRNA locus from the snails’ chemosynthetic symbionts (table 3). The *16S* rRNA gene of gill endosymbionts of two *A. adamantis* and five *I. nautilei* individuals was cloned prior to Sanger sequencing (Goffredi et al. 2007) with 24 colonies per individual. All PCR products were diluted in 40–50 μl of sterile water and purified with a Multiscreen HTS PCR 96 vacuum manifold system (Millipore Corp., Billerica, MA). PCR products were sequenced bidirectionally on an ABI 3130XL sequencer with BigDye Terminator v3.1 chemistry (Life Technologies Corp., Carlsbad, CA) and primers used in PCR. Samples from American Samoa were added late to the study and were only included in phylogenetic analyses of the symbionts. Forward and reverse sequences were trimmed, merged, and manually curated in Geneious Prime (http://www.geneious.com; last accessed July 24, 2020). Heterozygous sites at nuclear loci were phased with Phase v2.1.1 (Stephens and Donnelly 2003) as in Breusing et al. (2015).

**Table 3.** PCR Primers and Methods for Gene Loci Used in *Alviniconcha* spp. and *Ifremeria nautilei*.

Locus	Product	Primers	Reference	Methods	Length
Mitochondrial					
*COI*	Cytochrome-*c*-oxidase subunit-I	HCO/LCO	Folmer et al. (1994)	^a^	∼650∼1,200
		COIF/R	Nelson and Fisher (2000)		
*mt-16S*	16S mitochondrial RNA	16SAR/BR	Palumbi (1996)	Fast PCR	∼500
*mt-12S*	12S mitochondrial RNA	12SF/R	Kocher et al. (1989)	Fast PCR	∼440
Nuclear					
*28S-D1*	28S ribosomal RNA subunit-D1	28SD1F/R	Colgan et al. (2000)	Fast PCR	∼350
*28S-D6*	28S ribosomal RNA subunit-D6	28SD6F/R	McArthur and Koop (1999)	Fast PCR	∼450
*18S*	18S ribosomal RNA	18S1F/4R	Giribet et al. (1996)	Fast PCR	∼550
*H3*	Histone-3	H3F/R	Colgan et al. (2000)	Fast PCR^b^	∼330
*ATPSa*	ATP synthetase subunit a	ATPSaF/R	Jarman et al. (2002)	Fast PCR	∼410–750
*ATPSb*	ATP synthetase subunit b	alvATPSbF/R	This study^c^	Fast PCR	∼400–500
*EF1a*	Elongation factor 1 a	EF1F/R	Colgan et al. (2007)	Fast PCR	∼400
Symbiont					
*16S*	Symbiont 16S rRNA	27F/1492R	Lane (1991)	^d^	∼1,500

a PCR program: 95 °C/10 min; 35 × [94 °C/1 min, 55 °C/1 min; 72 °C/1 min], extension at 72 °C/7 min.

b Touchdown and Fast PCR: Amplitaq Gold Fast PCR Master Mix, UP (Life Technologies Corp.) and the protocol for the *Taq* supplied by the manufacturer of the Veriti thermal cycler with an annealing temperature at 50 °C (Life Technologies Corp.).

c Modified after Jarman et al. (2002), ATPSb-AlviniF: 5′-TCAGCCTCACCGATGACACCT-3′ and ATPSb-AlviniR: 5′-CRGGGGGYTCRTTCATCT-3′.

d PCR program: 95 °C/10 min; 25 × [92 °C/1 min, 53 °C/1 min; 72 °C/1 min], extension at 72 °C/7 min.

### Haplotype Networks, Genetic Structure, and Admixture

Haplotype networks for two mitochondrial genes (*COI*, *12S*) and four nuclear genes (*H3*, *EF1a*, *ATPSa*, and *ATPSb*) were constructed in Popart v1.7 (http://popart.otago.ac.nz/; last accessed July 24, 2020) with the median-joining algorithm as in Breusing et al. (2019).

Structure v.3.5 (Pritchard et al. 2000) was applied to investigate the number of genetic clusters *K* in the total *Alviniconcha* sample set and to test for contemporary admixture in the co-occurring species *A. boucheti*, *A. kojimai*, and *A. strummeri*. Analyses were run for all six species together as well as for each species separately using 1) mitochondrial and nuclear data and 2) nuclear data only. Allelic information was provided as a number code. Inter-specific Structure analyses were based on five (*COI*, *H3*, *EF1a*, *ATPSa*, and *ATPSb*) and four genetic loci (*H3*, *EF1a*, *ATPSa*, and *ATPSb*), respectively. The same genetic markers were used in intra-specific analyses, except that we excluded the *H3* locus given that it was essentially monomorphic within *Alviniconcha* populations. Model runs and settings for Structure generally followed Breusing et al. (2016) with ten repetitions for each *K* between 1 and 8 for inter-specific analyses and between 1 and 6 for intra-specific analyses. We subsequently used Clumpak v.1.1 (Kopelman et al. 2015) to integrate results across independent runs for each *K* value and determine the optimal clustering solution. We initially used the Δ*K* method (Evanno et al. 2005) to objectively infer the number of genetic clusters in the data set. However, as this method is biased toward identifying the uppermost hierarchical level of population structure (i.e., lowest parsimonious *K* value) (Cullingham et al. 2020), we visually inspected all *K* plots for biologically meaningful structuring and chose the highest *K* value where genetic subdivision could be observed (Meirmans 2015).

To distinguish hybridization from incomplete lineage sorting in the sympatric species *A. boucheti*, *A. kojimai*, and *A. strummeri*, we performed two ABBA–BABA tests on the concatenated nuclear gene alignments of 90 snail individuals using HybridCheck (Ward and van Oosterhout 2016). Gene flow was assessed between 1) *A. boucheti* (P3) and *A. kojimai*/*A. strummeri* (P1 + P2) and 2) *A. strummeri* (P3) and *A. kojimai*/*A. hessleri* (P1 + P2), with *A. adamantis* and *A. boucheti* as outgroups, respectively. Sequences from *I. nautilei* were not used because of missing data. Jackknife sampling was performed on four blocks of 305 bp (i.e., the approximate size of each nonlinked locus).

### Fossil-Calibrated Phylogeny of the Abyssochrysidae

All *Alviniconcha* species were included in a fossil-calibrated phylogenetic reconstruction of the Abyssochrysidae with the program Beast v.2.5.7 (Bouckaert et al. 2014). Fossil calibrations included the divergence of *Neptunea amianta* and *N. antiqua*, which have probably been separated since the earliest appearance of the genus in the Late Eocene, 33–37 Ma (Amano 1997), as well as *Provanna* and *Desbruyeresia*, which are known from Cenomanian (Cretaceous) seep deposits in Japan, 93–100 Ma (Kaim et al. 2008). These estimates can be considered a minimum age of the split between the genera. As sequences for the nuclear DNA markers *EF1a*, *ATPSa*, and *ATPSb* could not be obtained for fossil samples, only ribosomal and mitochondrial markers plus the nuclear *H3* locus were used for analysis (table 3). New sequences for all species of *Alviniconcha* from several localities were added to existing alignments from Johnson et al. (2010, 2015) with default parameters using Muscle executed in Geneious Prime (http://www.geneious.com; last accessed July 24, 2020). Fragments of the *16S* mtRNA and *12S* mtRNA loci that had been previously excluded due to alignment ambiguities with more distantly related taxa were informative and therefore included within the genera *Alviniconcha* and *Ifremeria*. For the rest of the Abyssochrysidae, these regions are represented as missing data. For each locus and species, the most common allele was chosen as representative sequence for phylogenetic reconstructions. JModelTest v2 (Darriba et al. 2012) was used to identify the best fitting evolutionary model for each gene and to partition the data for phylogenetic analyses. Concatenated gene trees were estimated based on a relaxed, lognormal clock with a Yule tree prior, a GTR + I + Γ substitution model as well as unlinked base frequencies and rates. The Markov chain Monte Carlo chains were run for 200 million generations, with parameters sampled every 5,000 generations. We used Tracer v1.7.1 (Rambaut et al. 2018) to determine if effective sample sizes were adequate for all parameters and if analyses had reached convergence. Node dates were calibrated as a normal distribution around the timing of genera splits.

A StarBeast v2.5.7 (Bouckaert et al. 2014) species tree was estimated including only the genera *Alviniconcha* and *Ifremeria* as replicate individuals for more distantly related taxa were missing. These estimated trees included at least one individual from each population per species or replicates from a single population. Species trees were calculated based on 1) the same loci used for the concatenated gene tree as well as 2) all available genetic markers (i.e., including the nuclear genes *EF1a*, *ATPSa*, and *ATPSb*). Phylogenetic reconstructions were performed with loci unlinked by partition, a GTR + I + Γ site model with empirical frequencies, an uncorrelated lognormal clock, an analytical population size integration model, and a Yule tree prior. Markov chain Monte Carlo analyses were run for 200 million generations, with a tree sampled every 10,000 generations, a preburnin of 1,000 and 10 initialization steps. All analyses were run multiple times and results assessed in Tracer, FigTree v1.4.4 (http://tree.bio.ed.ac.uk/software/figtree/; last accessed July 24, 2020), and Densitree v2.2.1 (Bouckaert 2010).

### Phylogenetic Analyses of Snail Endosymbionts

New *16S* rRNA endosymbiont sequences from the gills of *A. adamantis* and *I. nautilei* were compared against the NCBI nonredundant database. The new sequences were then aligned with Sina v1.2.11 (Pruesse et al. 2012) against the global Silva *16S* rRNA alignment with available *16S* rRNA endosymbiont sequences from GenBank for *Alviniconcha* species, *I. nautilei*, and other representative chemosynthetic taxa (Beinart et al. 2012, 2015). Phylogenetic trees for the Gammaproteobacteria and Campylobacteria symbionts were constructed with MrBayes v.3.2.7a (Altekar et al. 2004) under a GTR + I +  Γ substitution model in the Cipres Science Gateway v3.3 (Miller et al. 2010). Two sets of three heated chains and one cold chain were run for 1,100,000 generations and sampled every 100 generations. The first 100,000 generations were discarded as burnin. Reference sequences of *Nautilia lithotrophica* (NR_025497) and *Beggiatoa alba* (NR_041726) were used as outgroups for the Campylobacteria and Gammaproteobacteria trees, respectively. Ancestral endosymbiosis traits were identified with Mesquite v.2.75 (Maddison WP and Maddison DR 2011).

### High-Throughput 16S V4–V5 Amplicon Sequencing and Analysis

The *16S V4–V5* hypervariable region was investigated to assess symbiont phylotypic variation in relation to host species and geographic location. After overnight lysis in proteinase K, DNA from RNALater (Thermo Fisher Scientific, Inc.) preserved gill samples was extracted with the Zymo Quick DNA 96 Plus kit (Zymo Research, Inc.) and further cleaned with the ZR-96 Clean-up kit (Zymo Research, Inc.) following the manufacturer’s protocols. Barcoded amplicon libraries (515 samples and 62 negative controls) targeting a ∼375-bp fragment were prepared using the primer pairs 515F/926R (Walters et al. 2015) and sequenced on an Illumina MiSeq platform with a 2× 250-bp protocol at the Argonne National Laboratory (Lemont, IL). Host species identity was determined based on morphology (*Ifremeria*) or molecular barcoding of the mitochondrial *COI* gene (*Alviniconcha*).

After quality-checking the demultiplexed reads with FastQC v0.11.5 (Andrews 2010; http://www.bioinformatics.babraham.ac.uk/projects/fastqc/; last accessed July 24, 2020), we clipped adapters with Trimmomatic v0.36 (Bolger et al. 2014) and followed the Usearch v11 (Edgar 2010) pipeline for analysis. Reads were merged allowing lengths >300 bp, and then filtered applying a maximum error rate of 0.001, a quality threshold of 20, and a minimum length of 300 bp. The *fastx_uniques* and *unoise3* commands were used to dereplicate, denoise, and cluster the reads into ZOTUs, whereas the *otutab* command was used to generate the OTU table. Taxonomy was assigned to each ZOTU in Qiime2 (https://qiime2.org; last accessed July 24, 2020) with a Naïve Bayes classifier that we trained against the target *16S V4–V5* region using the Silva 132 99% reference database. Ambiguous annotations were resolved using BlastN searches (Altschul et al. 1990). Recent studies have shown that well-to-well contamination accounts on average for 2.37% of reads in high biomass samples that were processed at the Argonne National Laboratory (Minich et al. 2019). Consequently, for further analysis, we removed ZOTUs that had <2.37% abundance in a sample, ignored all ZOTUs that were not annotated as vent snail endosymbionts and we excluded all samples with <1,000 symbiont reads. To identify any unrecovered phylotype variation, we analyzed the filtered symbiont read set with the Oligotyping v2.0 method (Eren et al. 2013) based on 24 informative nucleotide positions. Only oligotypes with at least 2.37% abundance in a sample (–a), 100 supporting reads (–M), and occurrence in more than one individual (–s) were considered. We further excluded reads that had base quality scores <20 in the nucleotide positions of interest (–q).

Nonmetric multidimensional scaling and diversity analyses for the final symbiont oligotype data set were performed with the PhyloSeq package in R v3.5.2 (McMurdie and Holmes 2013; R Core Team 2018) based on weighted Unifrac distances on log-transformed read counts. We also performed partial Mantel tests using the Vegan package (Oksanen et al. 2019) to identify relationships between *Alviniconcha* host and symbiont genetic distances and geography based on Pearson’s product–moment correlations. Pairwise host genetic distances were calculated for mitochondrial *COI* sequences based on Kimura’s two-parameter model (Kimura 1980). For geographic distances, we determined the geodesics between vent sites using the Geosphere package (Hijmans 2019).

## Supplementary Material

Supplementary data are available at *Molecular Biology and Evolution* online.

## Supplementary Material

msaa177_supplementary_data

## Notes

New Sanger sequences for *Alviniconcha* and *Ifremeria* hosts and symbionts are available in GenBank under accession numbers MT131487–MT131781 (*COI*), MT130994–MT131149 (*12S*), MT146898–MT147385 (*H3*), MT148092–MT148633 (*ATPSa*), MT148634–MT149209 (*ATPSb*), MT147386–MT148091 (*EF1a*), and MT137388–MT137420 (*16S*). *16S* amplicon sequences have been deposited in the Sequence Read Archive under BioProject numbers PRJNA473256, PRJNA473257, PRJNA610289, and PRJNA610290.
