# Supplementary figures and images for: Allopatric and Sympatric Drivers of Speciation in Alviniconcha Hydrothermal Vent Snails
Source: Mol Biol Evol. 2020 Jul 13;37(12):3469–84. doi: 10.1093/molbev/msaa177 (PMC7743903; doi:10.1093/molbev/msaa177)

## A Mitochondrial and nuclear data

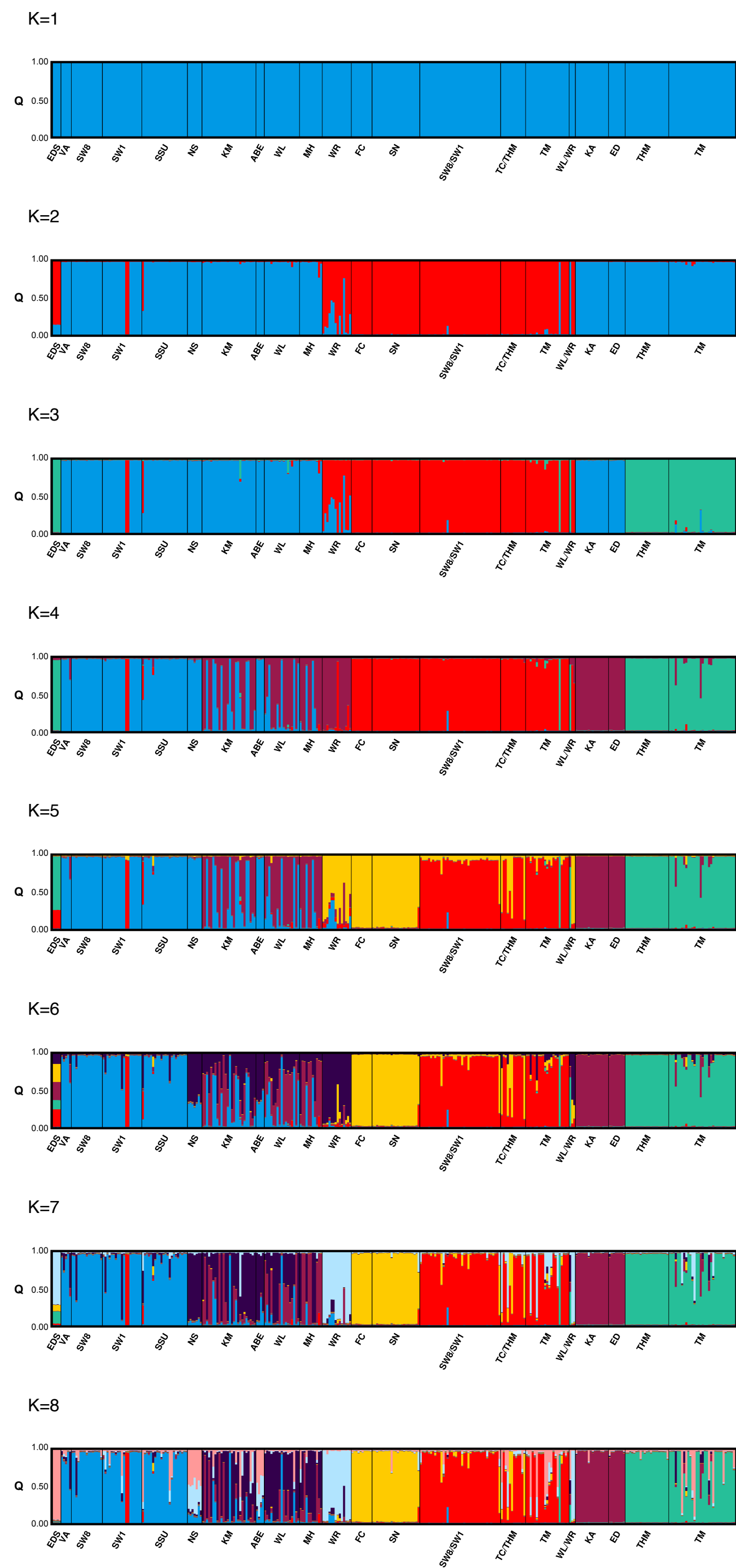

## B Nuclear data

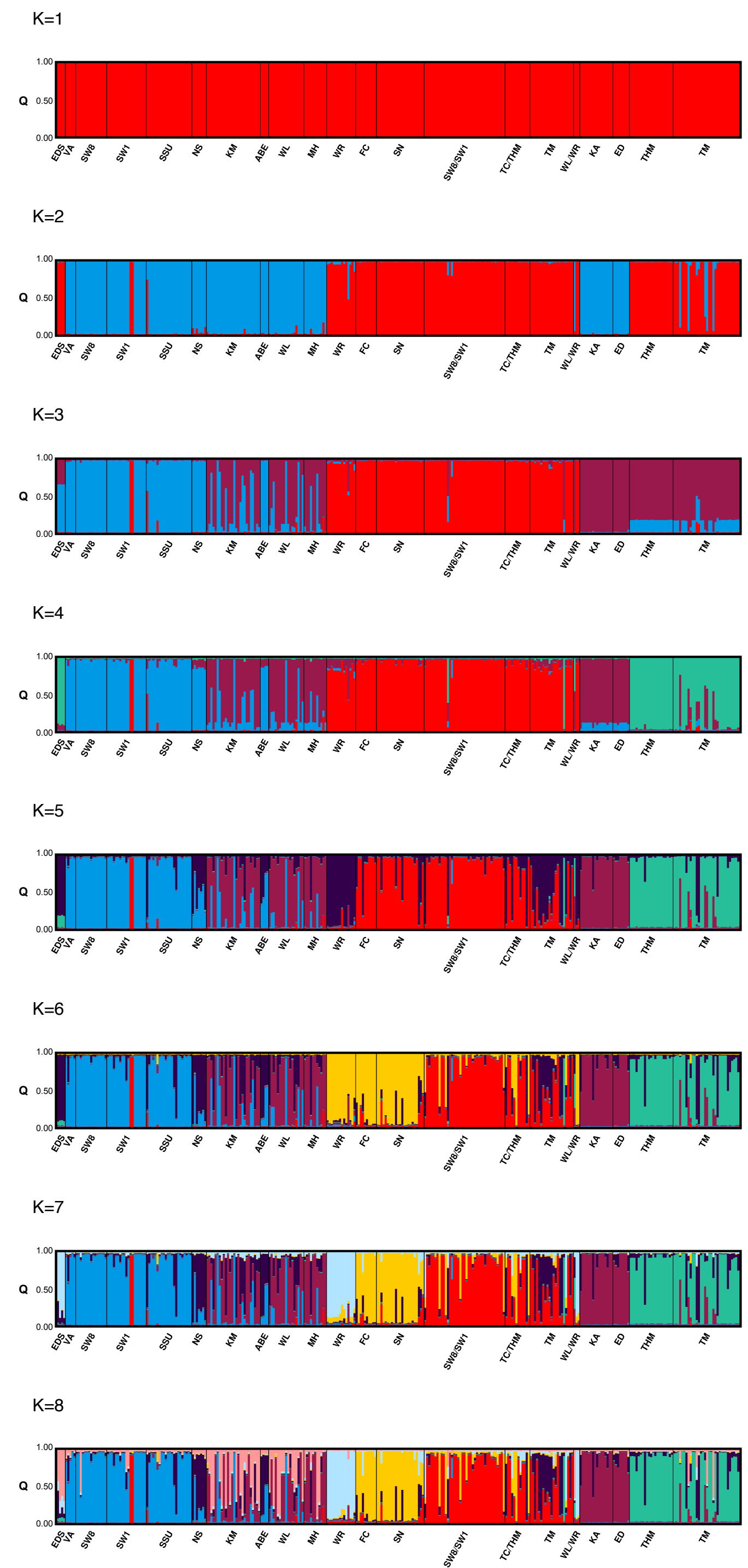

Supplement: msaa177_supplementary_data [file msaa177_supplementary_data.zip › FigS1.pdf]

**A** Mitochondrial and nuclear data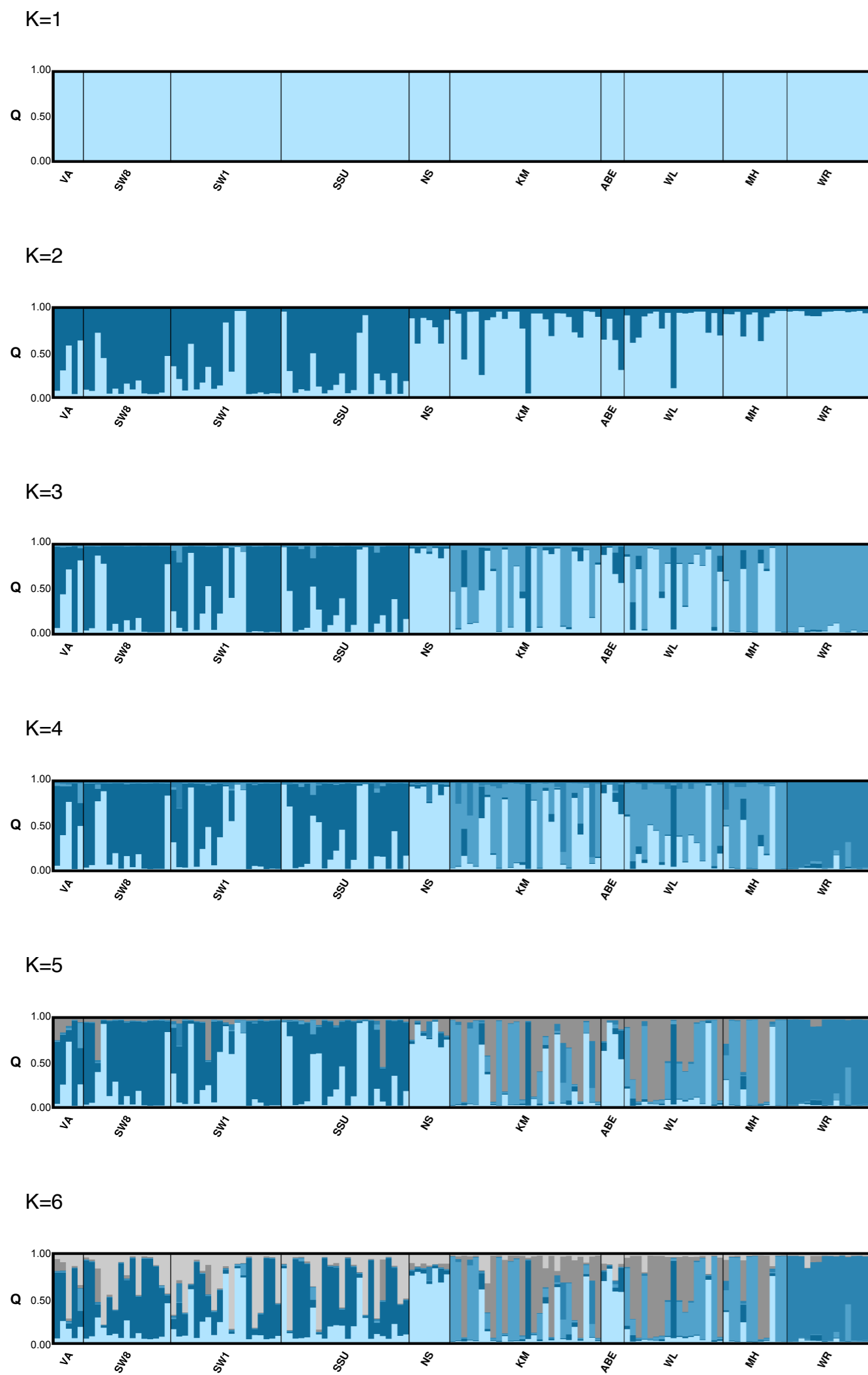**B** Nuclear data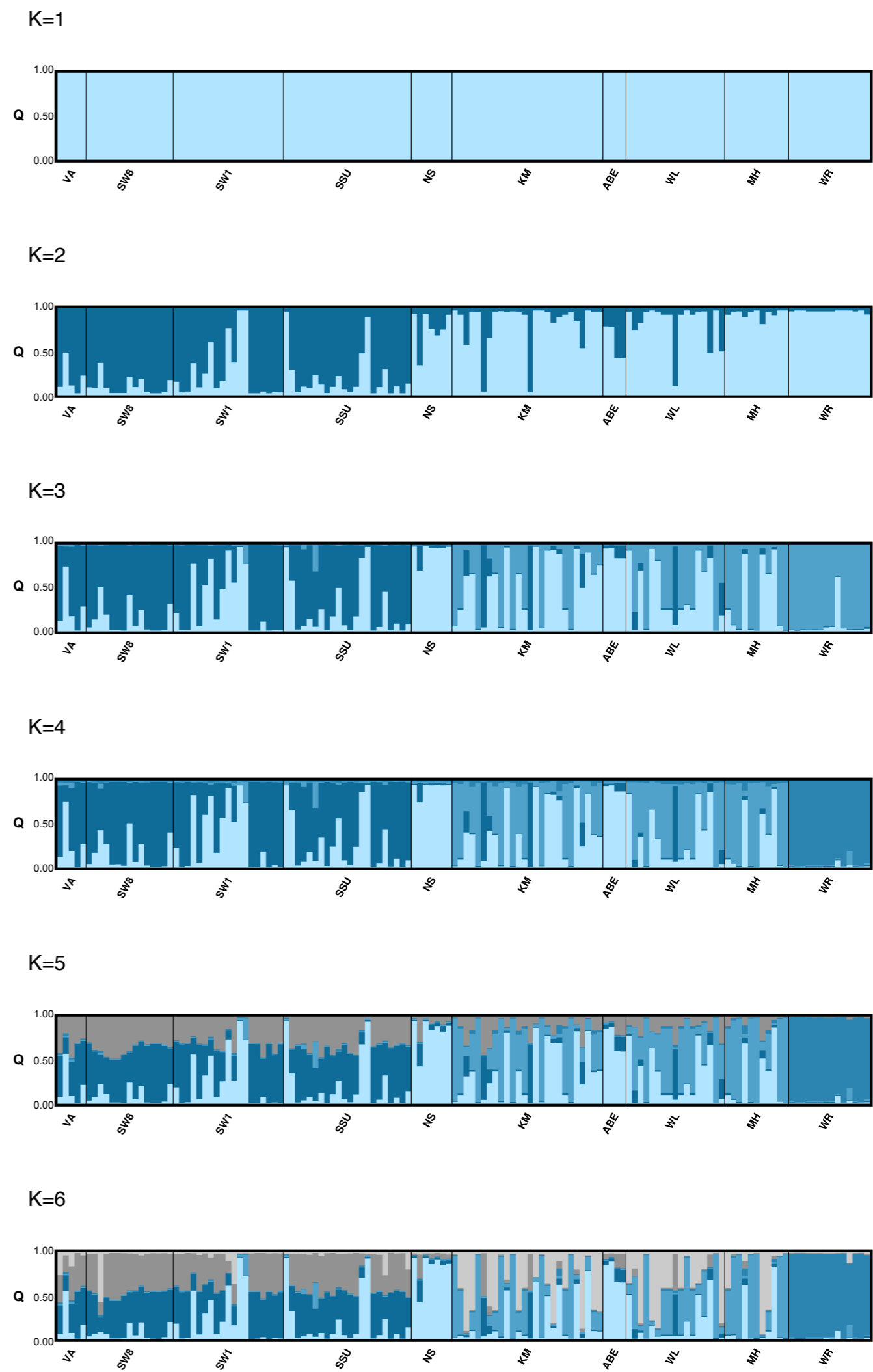

Supplement: msaa177_supplementary_data [file msaa177_supplementary_data.zip › FigS2.pdf]

**A** Mitochondrial and nuclear data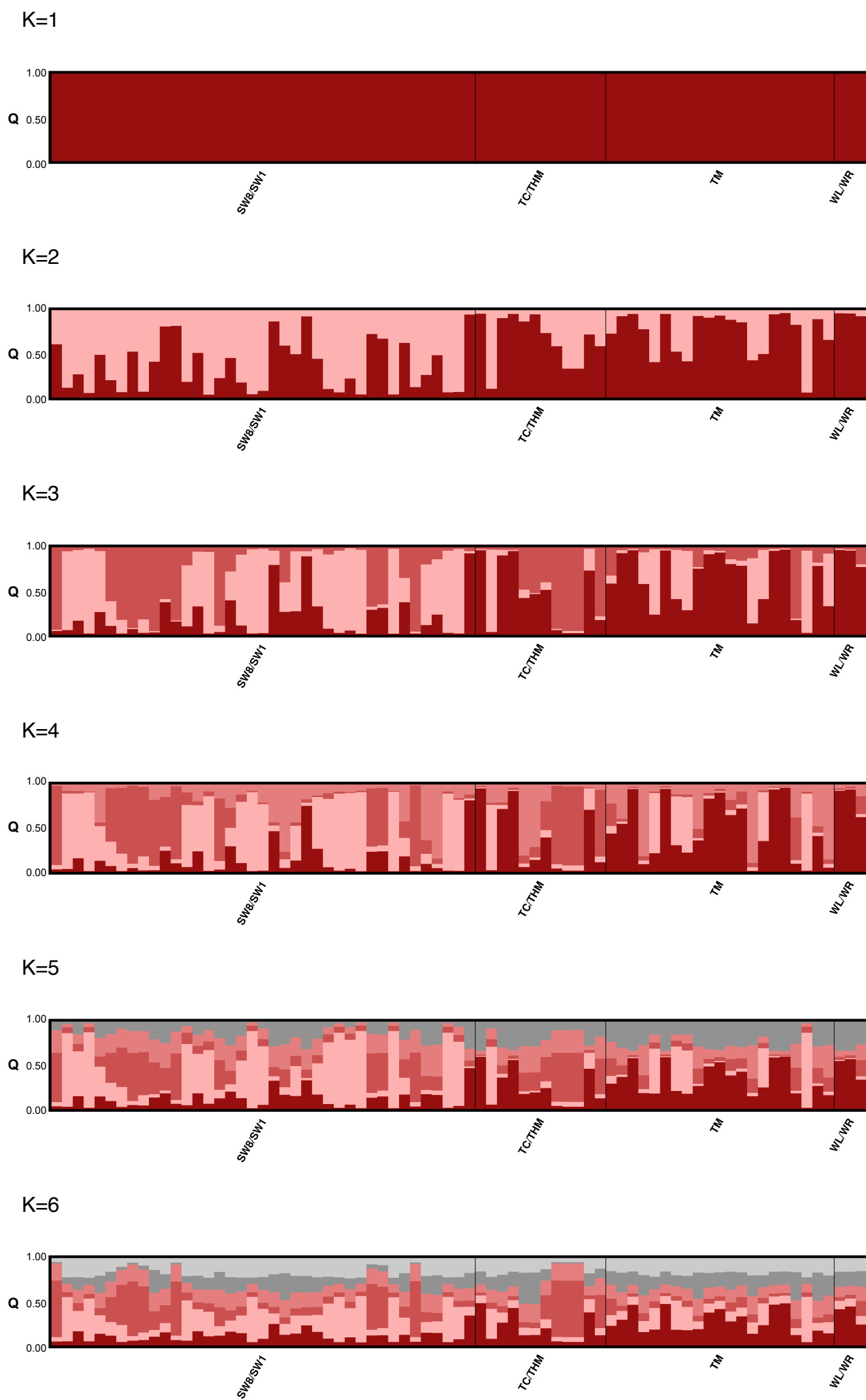**B** Nuclear data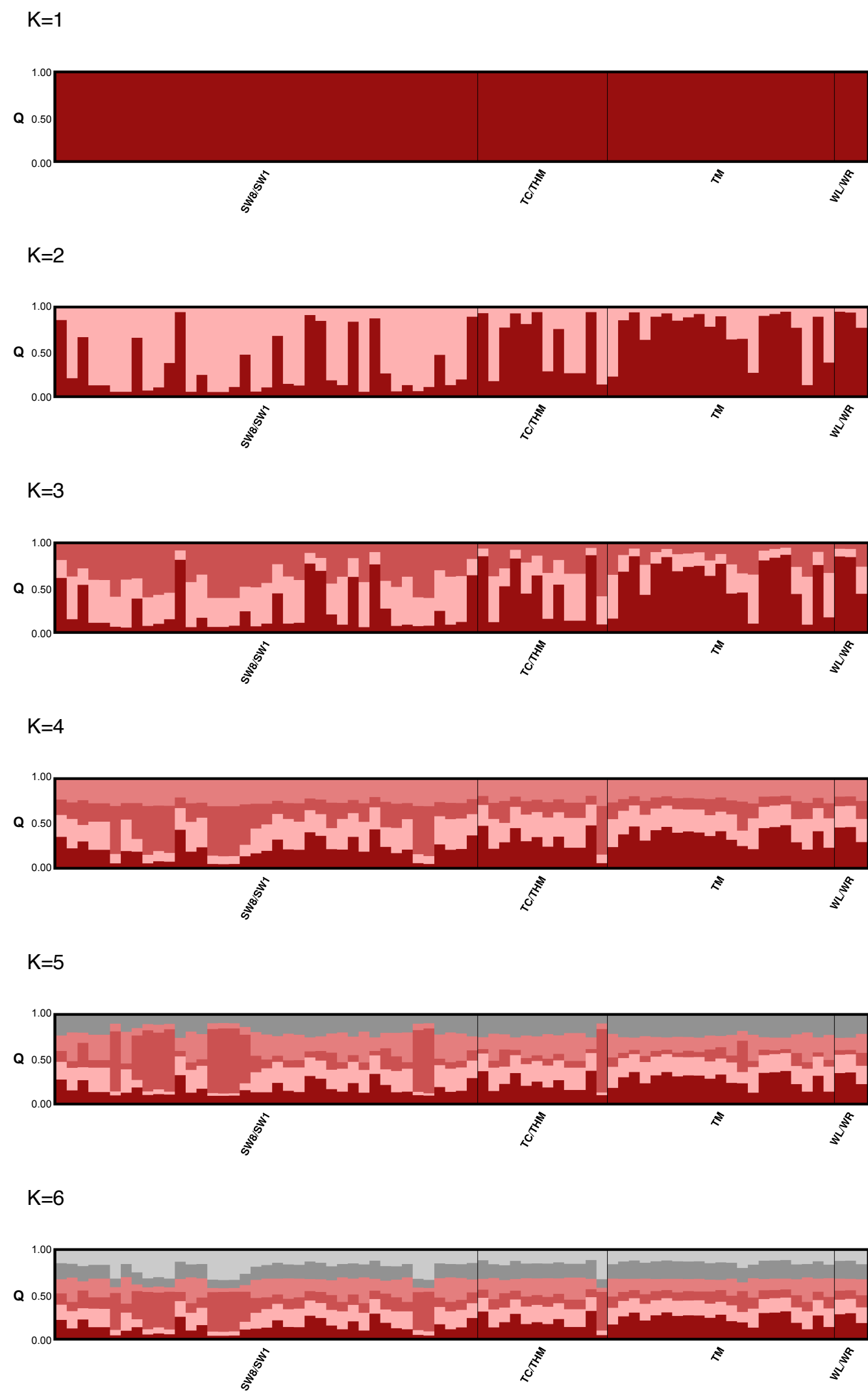

Supplement: msaa177_supplementary_data [file msaa177_supplementary_data.zip › FigS3.pdf]

**A** Mitochondrial and nuclear data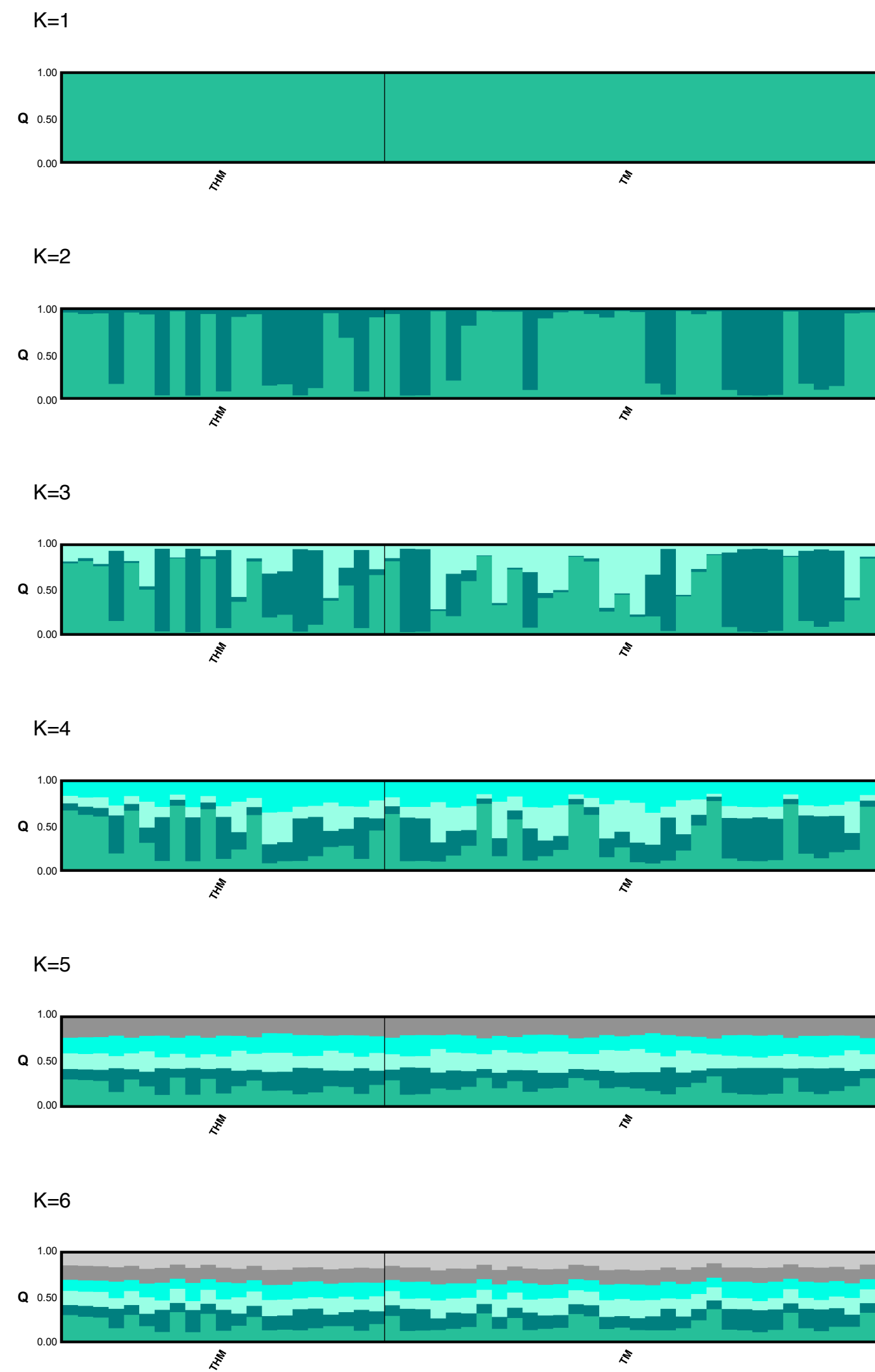**B** Nuclear data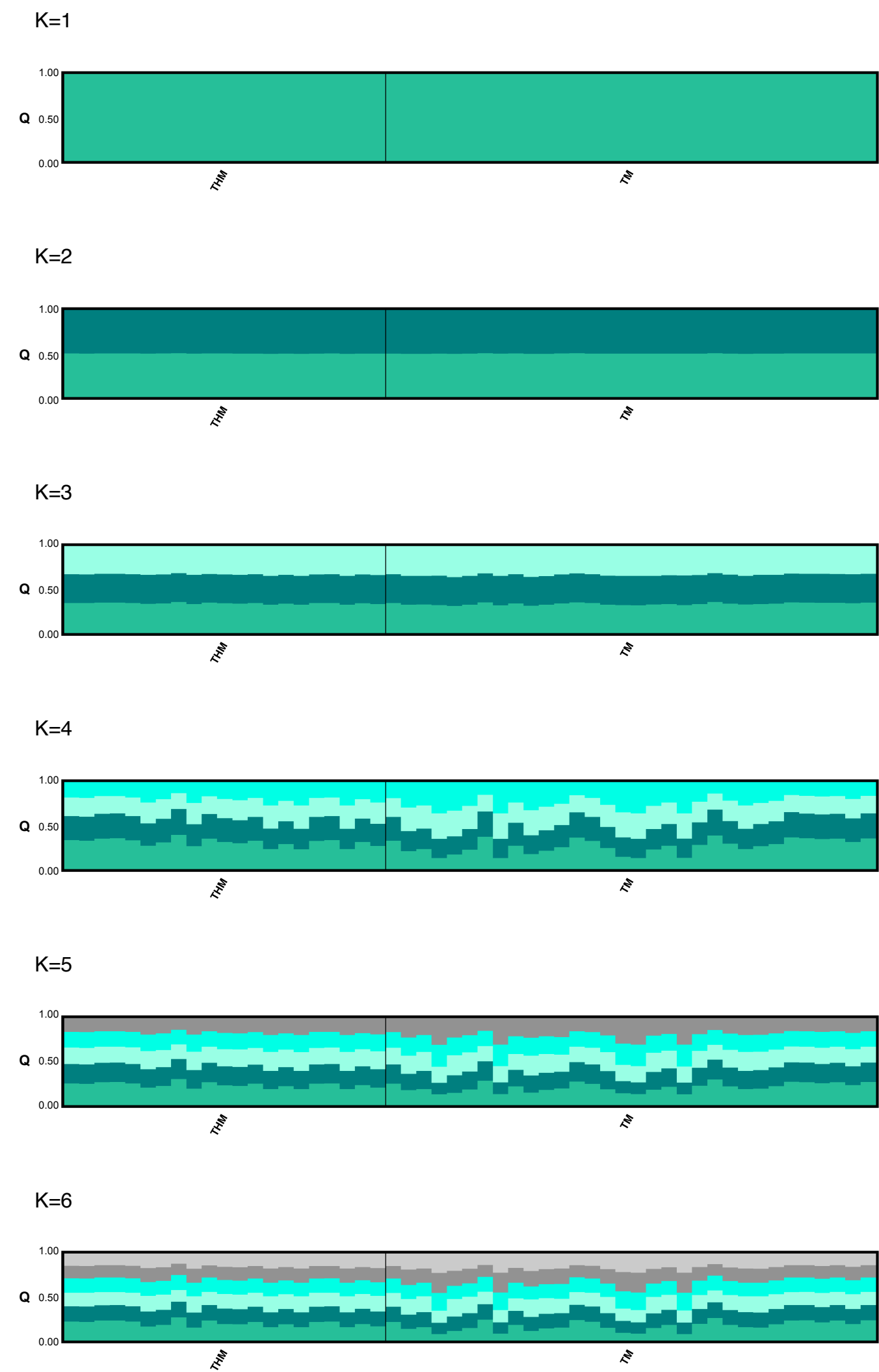

Supplement: msaa177_supplementary_data [file msaa177_supplementary_data.zip › FigS4.pdf]

**A** Mitochondrial and nuclear data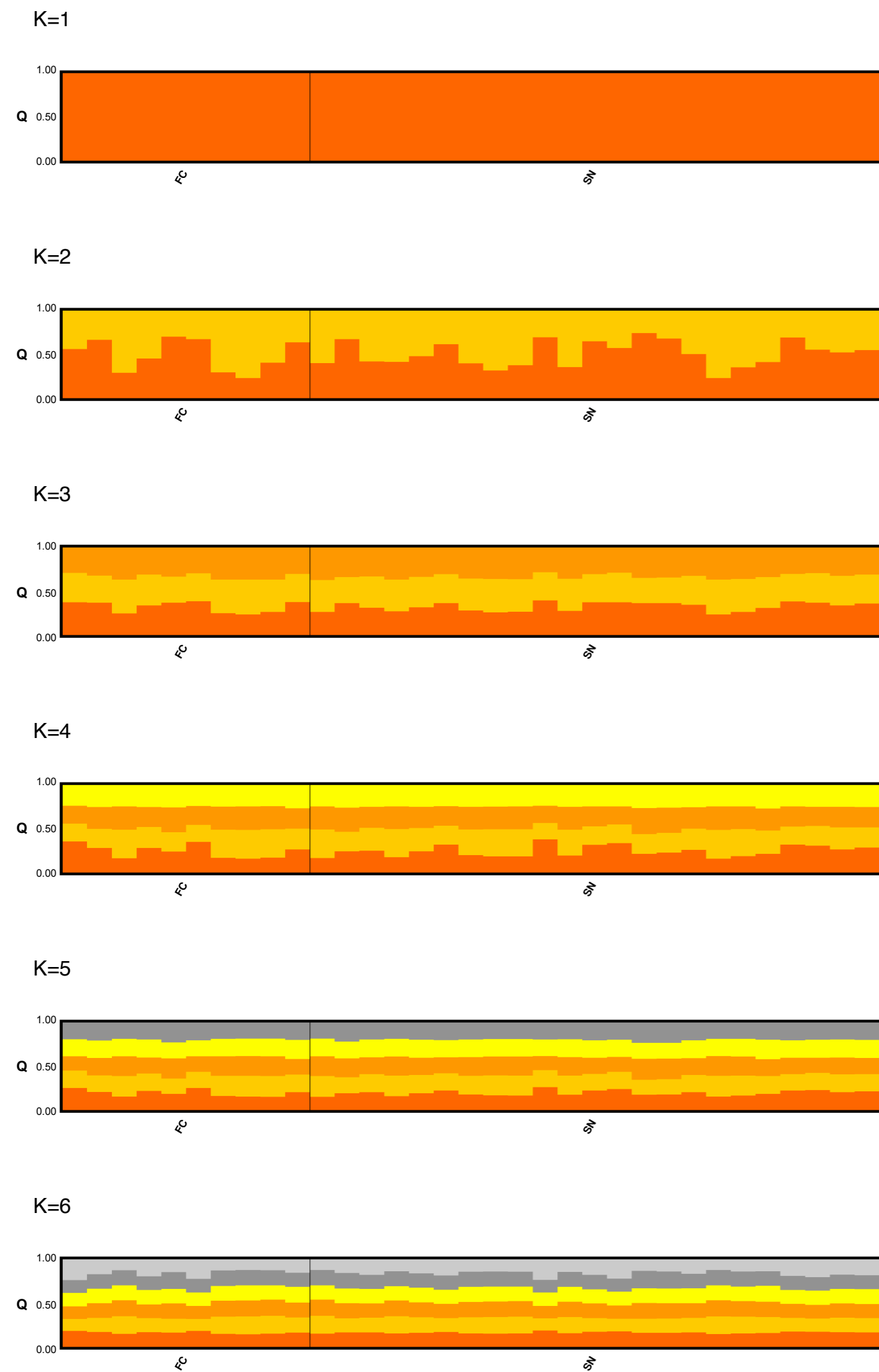**B** Nuclear data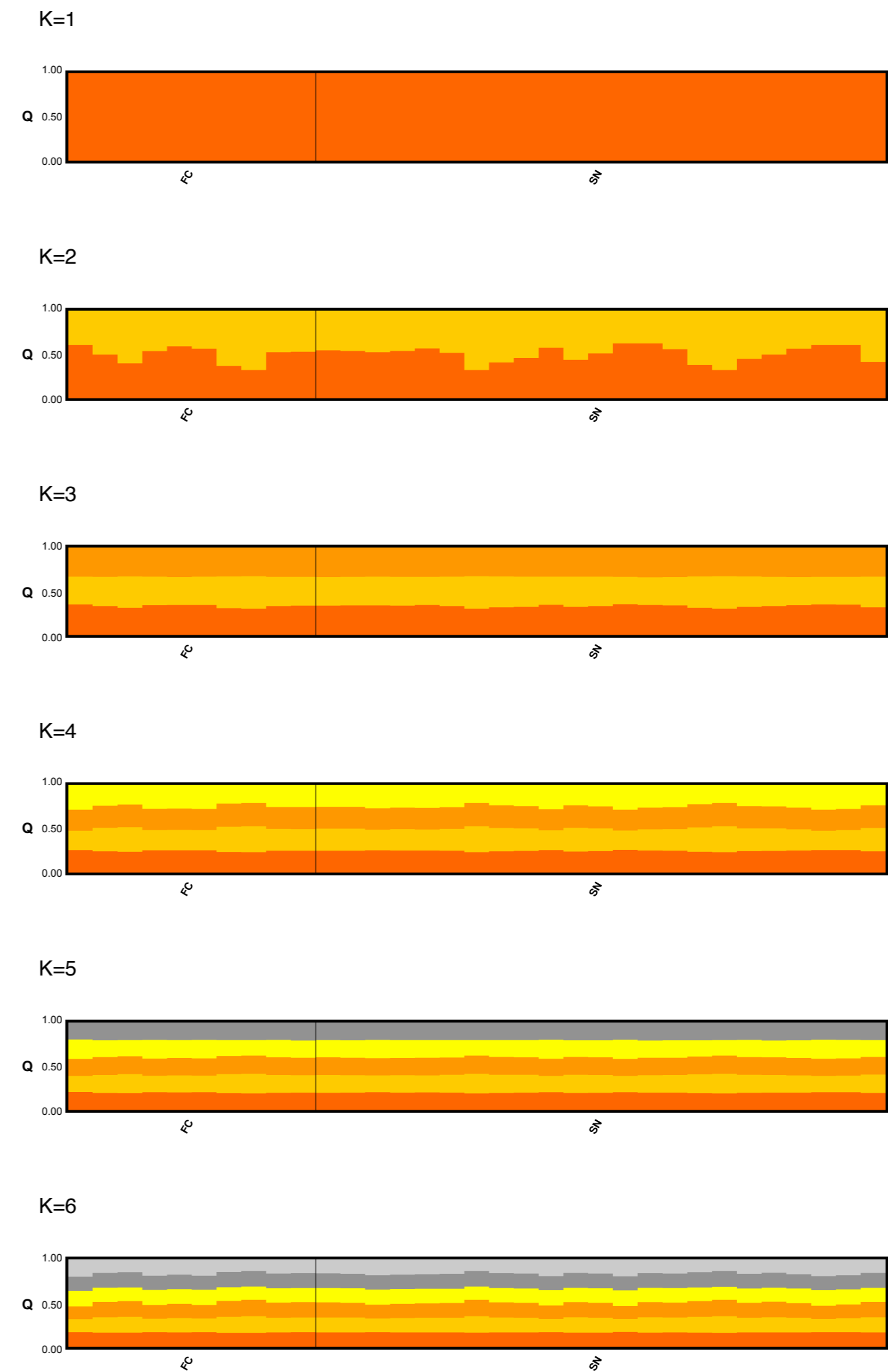

Supplement: msaa177_supplementary_data [file msaa177_supplementary_data.zip › FigS5.pdf]

**A** Mitochondrial and nuclear data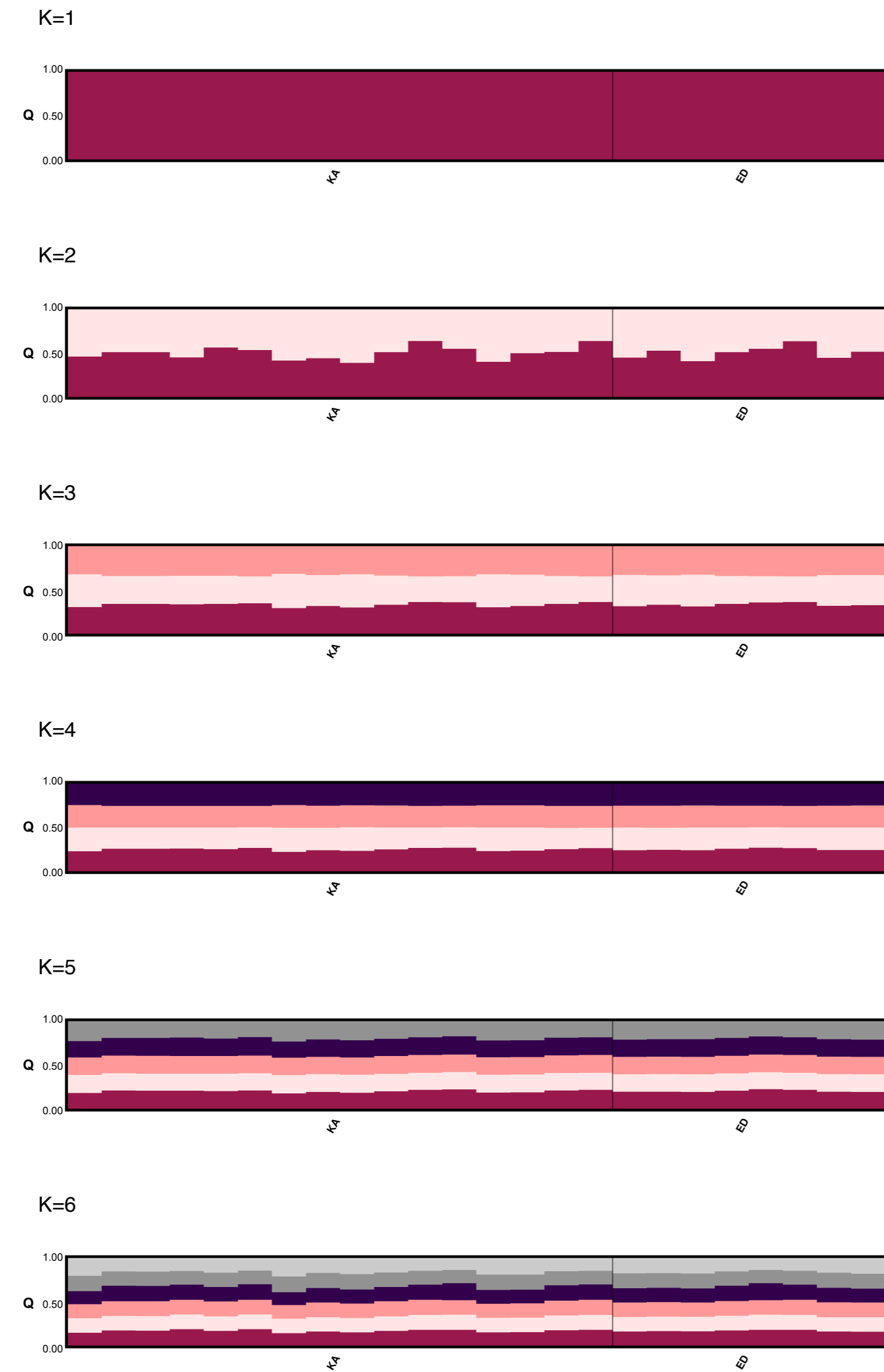**B** Nuclear data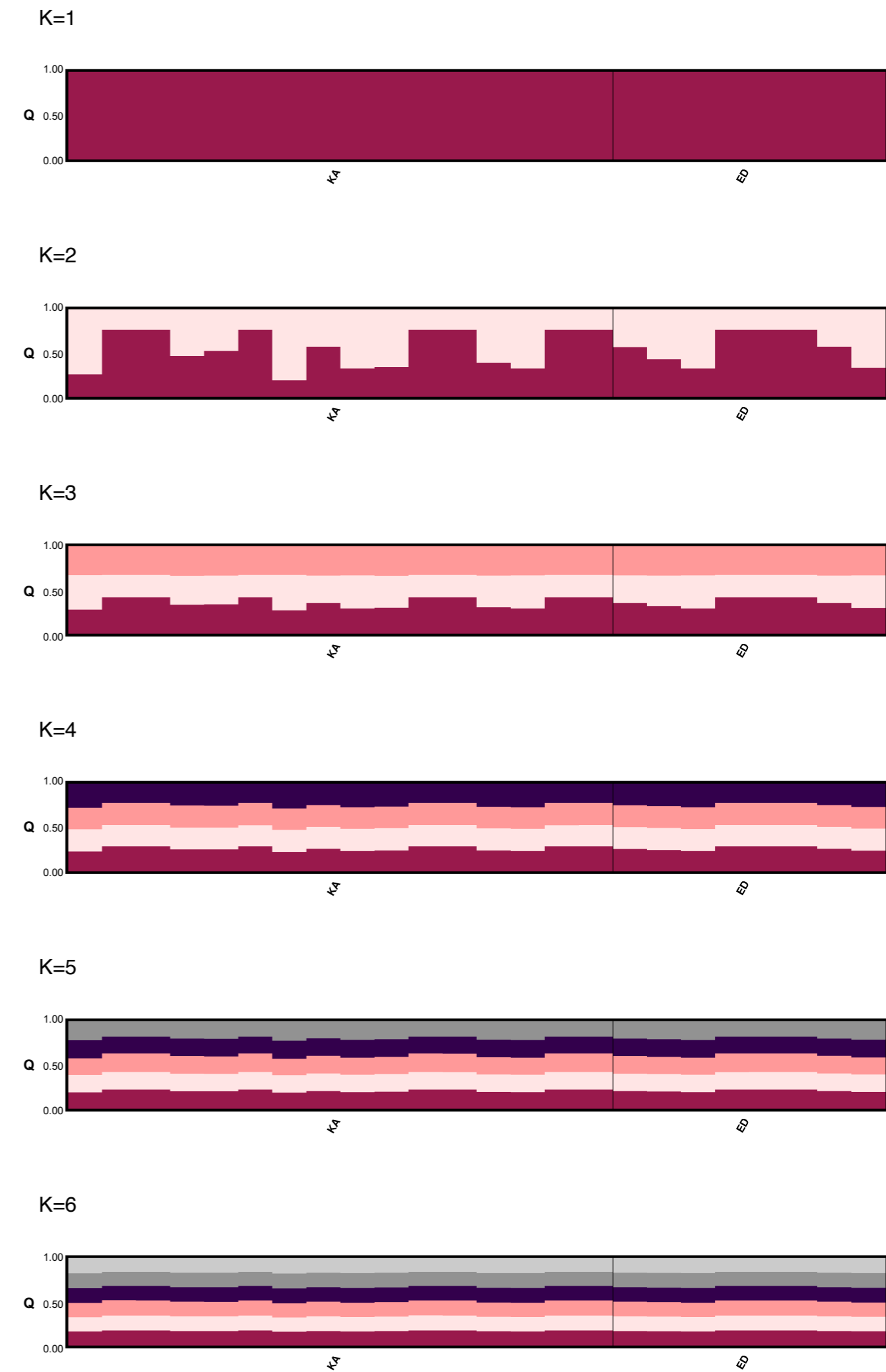

Supplement: msaa177_supplementary_data [file msaa177_supplementary_data.zip › FigS6.pdf]

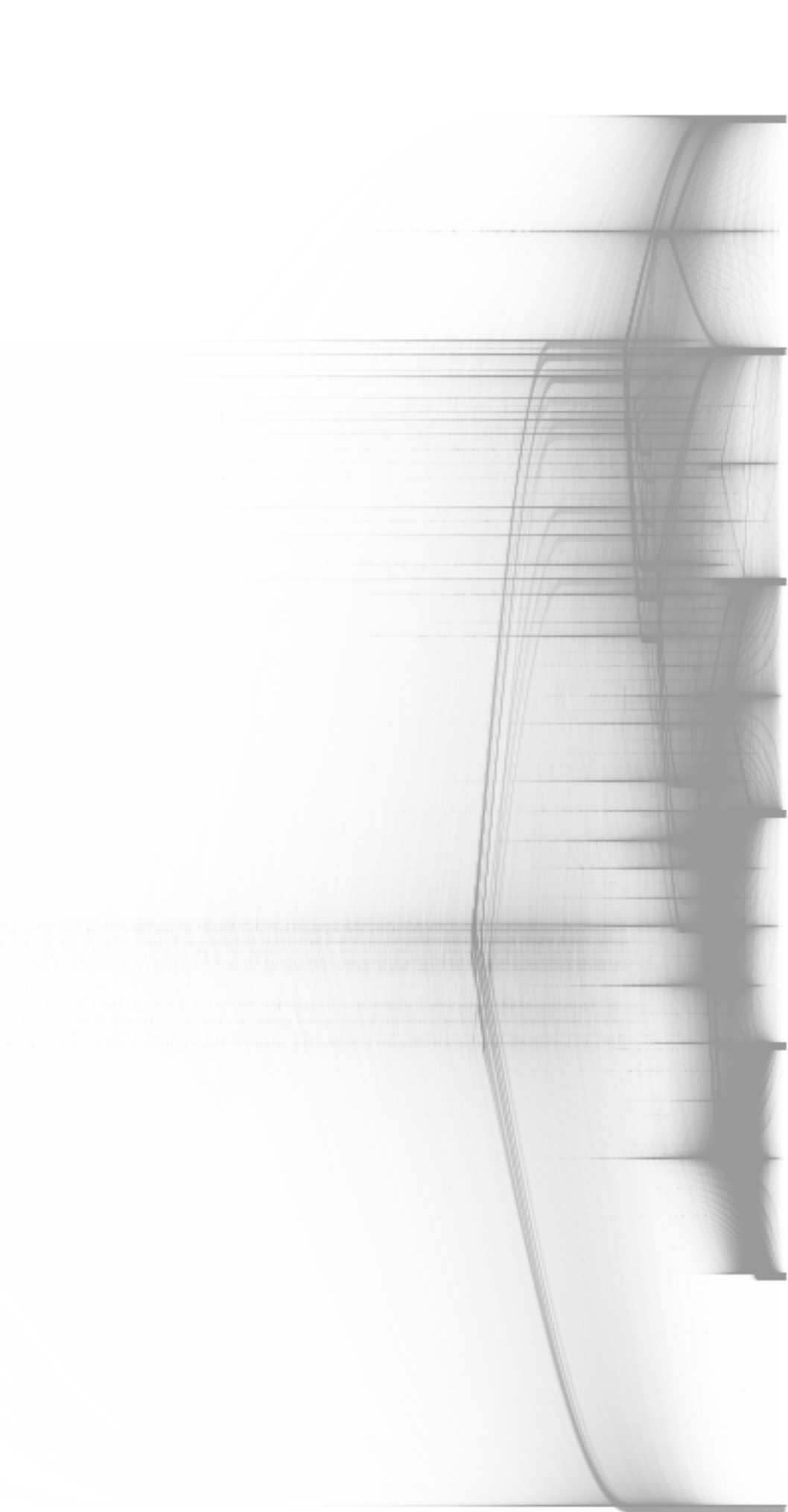

*A. adamantis*

*A. strummeri*

*A. marisindica*

*A. boucheti*

*A. kojimai*

*A. hessleri*

*I. nautilei*

Supplement: msaa177_supplementary_data [file msaa177_supplementary_data.zip › FigS7.pdf]
